# Beyond Pristine Layers: Engineering van der Waals MPX_3_ Materials (M: Transition Metal, X: Chalcogen) via Defects, Adsorption, and Intercalation

**DOI:** 10.1002/smsc.202500307

**Published:** 2025-08-20

**Authors:** Yuriy Dedkov, Elena Voloshina

**Affiliations:** ^1^ Center for Advanced Laser Techniques Institute of Physics Bijenička cesta 46 10000 Zagreb Croatia; ^2^ Division of Theoretical Physics Ruđer Bošković Institute Bijenička cesta 54 10000 Zagreb Croatia

**Keywords:** adsorption, defects, intercalation, trichalcogenides

## Abstract

Recent breakthroughs and achievements in the studies of 2D materials have led to the increased attention to the respective van der Waals parent compounds. Herein, the class of layered materials—so‐called transition metal phosphorus trichalcogenides (MPX_3_; M: transition metal, X: chalcogen)—has been recently investigated extensively due to the diversity in their properties depending on the M/X combination. Moreover, further studies demonstrate the large tunability of the electronic, optical, and magnetic properties of these materials using different methods, like defects’ engineering, alloying, adsorption, or intercalation. In the present review, several discussed approaches are focused on, highlighting the mechanisms leading to the properties’ modifications and drawing perspectives on the further studies and developments in this class of materials.

## Graphical abstract

This review highlights specific examples, demonstrating how the electronic and magnetic properties of transition metal phosphorus trichalcogenides (MPX_3_) can be systematically modified. Key approaches—including defect engineering, adsorption, alloying, and intercalation—are examined in detail. Finally, the review outlines future research directions that may enable the discovery of new phenomena and broaden the application landscape of MPX_3_ materials.

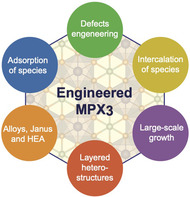

## 1 Introduction

The increasing demand for low power consumption and miniaturization of functional elements in electronic and optical devices drives the search for new low‐dimensional materials and systems—ideally 2D or 1D—that could serve as novel electrodes or insulating layers in such applications.^[^ ^1^, ^2^, ^3^ ^]^ Graphene, as a natural first choice among 2D materials due to its ease of isolation from bulk graphite or highly oriented pyrolytic graphite,^[^ ^4^ ^]^ has been intensively studied since the discovery of its remarkable transport properties in 2005.^[^ ^5^, ^6^, ^7^ ^]^ Subsequent experimental and theoretical investigations have revealed numerous fascinating properties of graphene, including ambipolar electron and hole transport,^[^ ^8^ ^]^ the quantum Hall effect at room temperature,^[^ ^9^ ^]^ substrate‐protective capabilities,^[^ ^10^, ^11^ ^]^ and long‐distance spin transport,^[^ ^12^, ^13^ ^]^ among others. Studies on bilayer and multilayer graphene have further shown that these systems provide a versatile platform for exploring exotic physical phenomena such as bandgap tuning^[^ ^14^ ^]^ and twist‐angle‐dependent superconductivity.^[^ ^15^, ^16^ ^]^ Despite these intriguing features, several limitations—such as graphene's zero bandgap, lack of intrinsic magnetic ordering, and challenges in achieving high material quality—hinder the practical application of single‐, bi‐, and multilayer graphene in advanced technologies. These limitations underscore the need for new mono‐ and multiatomic layered materials. This pursuit has recently led to the isolation and in‐depth exploration of the electronic, optical, and magnetic properties of alternative 2D materials, including silicene, phosphorene, and transition metal mono‐, di‐, and trichalcogenides.^[^ ^17^, ^18^, ^19^, ^20^ ^]^

Among the various 2D and layered materials, transition metal phosphorus trichalcogenides (TMTs)—represented by the structural formula MPX_3_ (where M is a transition metal, P is phosphorus, and X is a chalcogen) —have attracted growing interest. Their properties have been comprehensively reviewed in several recent publications.^[^ ^20^, ^21^, ^22^, ^23^ ^]^ Owing to the diversity of their electronic and magnetic structures across different MPX_3_ compounds, a wide range of potential applications has been proposed. These include low‐dimensional ferroelectricity,^[^ ^24^, ^25^ ^]^ their use in Li‐ and Mg‐ion batteries,^[^ ^26^, ^27^ ^]^ water splitting technologies,^[^ ^28^, ^29^ ^]^ and hydrogen storage.^[^ ^30^, ^31^ ^]^

In general, MPX_3_ compounds are wide‐bandgap semiconductors in which bandgaps depend on the specific combination of M and X atoms.^[^ ^20^, ^32^ ^]^ This family includes materials that exhibit various magnetic configurations of M^2+^ ions in their antiferromagnetic (AFM) ground states. The magnetic interactions between neighboring M^2+^ ions arise from a competition between direct AFM exchange and indirect ferromagnetic (FM) exchange mediated via the chalcogen atoms. In most studied MPX_3_ systems, the AFM interaction dominates, determining the ground magnetic state. However, both theoretical predictions and experimental confirmations have shown that the magnetic ground state can be tuned—either by suppressing the direct AFM exchange (e.g., through alloying) or by enhancing the indirect FM exchange (e.g., via electron or hole doping). Furthermore, the electronic structure of these materials can be engineered to adjust the bandgap and induce novel states, such as massless Dirac fermions in doped compounds.

**Figure 1:**
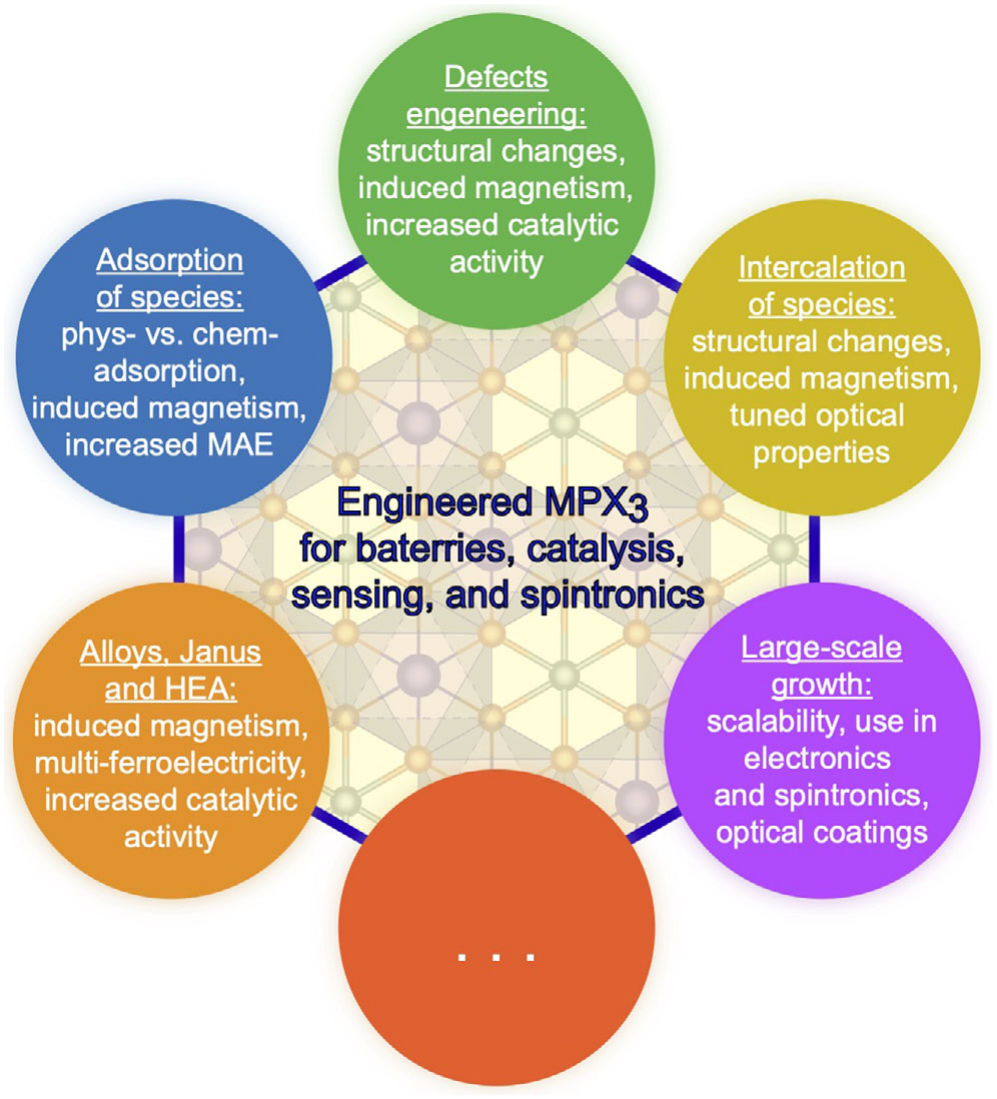
Strategies currently used to modify the properties of vdW MPX_3_ materials for their applications in various fields, highlighting several modified or induced properties.

Given the broad range of potential applications and tunable properties of MPX_3_ materials, this review focuses on specific examples illustrating how their electronic and magnetic structures can be modified in a controlled manner (**Figure** **1**). We highlight several key approaches—such as defect engineering, adsorption, alloying, and intercalation—that have been successfully employed to tailor their properties. Both theoretical and experimental advances are discussed, with an emphasis on representative case studies. Finally, we outline future directions that may enable the discovery of new phenomena and further expand the application space of MPX_3_ materials.

## 2 Ground State Properties of Bulk MPX_3_

The synthesis methods and various properties of MPX_3_ materials have been comprehensively reviewed in several recent publications.^[^ ^20^, ^21^, ^22^, ^23^ ^]^ Here, we briefly summarize only the aspects most relevant to the subsequent discussions.

**Figure 2:**
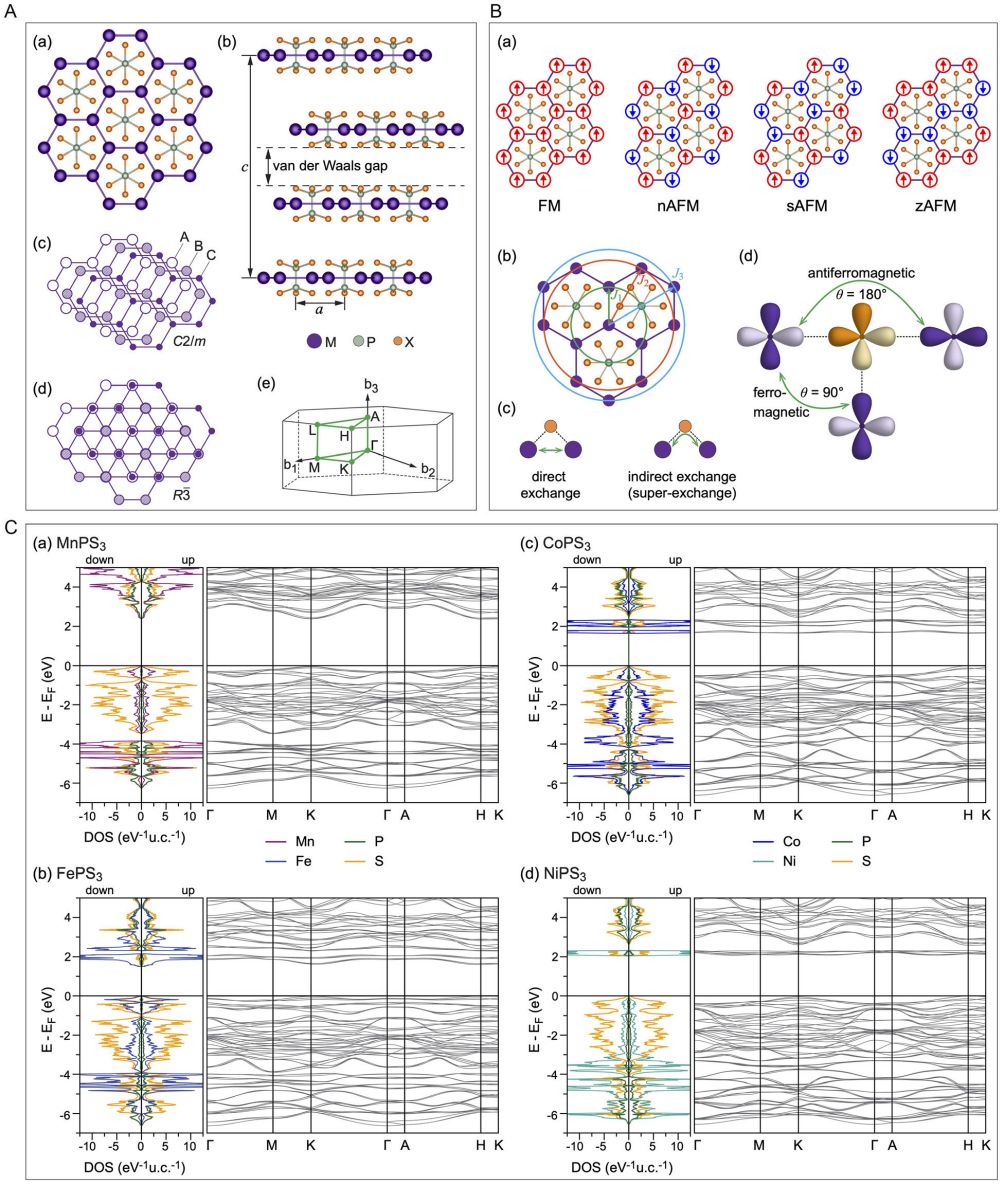
A) Crystallographic structure of bulk MPX_3_ crystals: a) top view of a single MPX_3_ layer; b) crystal structure of 3D MPX_3_; c,d) different stackings in bulk MPX_3_ corresponding to C2/m and $R\bar{3}$ space groups, respectively. For simplicity, only M‐ions are shown. Atoms of different layers are shown with spheres of different size and style; e) Brillouin zone for 3D MPX_3_ in the hexagonal representation. B) Magnetic structures of single MPX_3_ layers: a) four different magnetic configurations of 2D MPX_3_: ferromagnetic (FM), Néel antiferromagnetic (nAFM), stripy antiferromagnetic (sAFM), and zigzag antiferromagnetic (zAFM); b) the Heisenberg coupling parameters $J_{1}$, $J_{2}$, and $J_{3}$ in the MPX_3_ lattice; c) schematic representation of direct (M–M) and indirect superexchange (M–X–M) interactions, respectively; d) schematic representation of the FM and AFM coupling between M‐ions via ligand according to the Goodenough–Kanamori rules. C) Calculated atom projected DOS and band structures for bulk MPX_3_ crystals: a) MnPS_3_; b) FePS_3_; c) CoPS_3_; d) NiPS_3_. Reproduced with permission.^[^ ^20^ ^]^ Copyright 2023, IOP Publishing.

**Figure 3:**
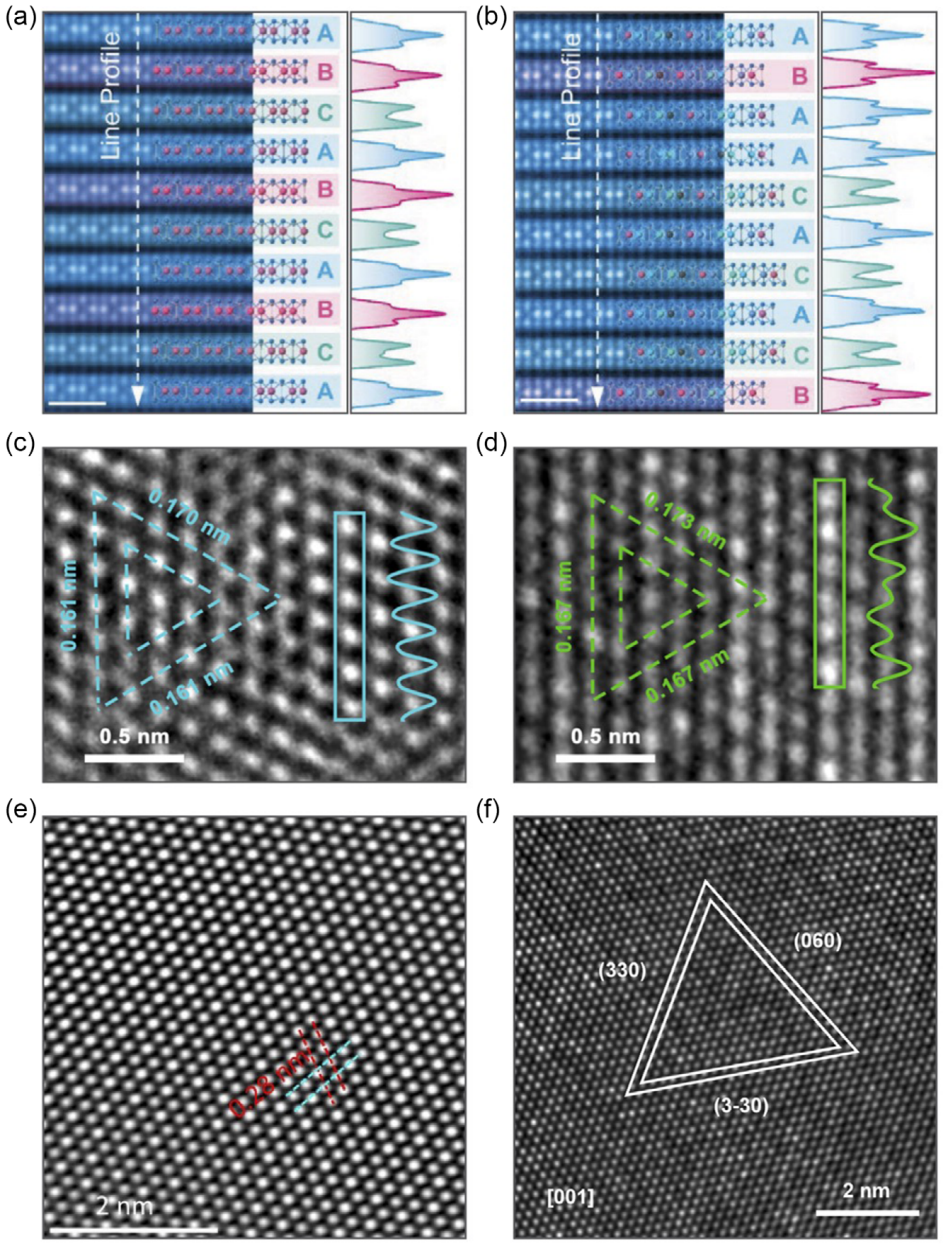
a,b) Cross‐sectional scanning TEM annular dark‐field images showing an ordered ABC stacking sequence in FePS_3_ (left panel) and an aperiodic stacking order in FeMnNiVZnPS_3_ (right panel). Corresponding intensity line profiles are also presented. Reproduced with permission.^[^ ^33^ ^]^ Copyright 2024, John Wiley and Sons. c,d) High‐resolution scanning TEM images and the respective intensity profiles for CoPS_3_ (left panel) and Co_0.6_(VMnNiZn)_0.4_PS_3_ (right panel). Corresponding intensity line profiles are also presented. Reproduced with permission.^[^ ^34^ ^]^ Copyright 2022, American Chemical Society. e,f) High‐resolution TEM images of NiPS_3_ (left panel) and NiFePS_3_ (right panel). Reproduced under the terms of the Creative Commons Attribution 4.0 license.^[^ ^35^ ^]^ Copyright 2023, The Authors. Published by IOP Publishing. Reproduced with permission.^[^ ^36^ ^]^ Copyright 2022, John Wiley and Sons.

MPX_3_ layered crystals are usually arranged in the C2/m and $R\bar{3}$ space groups for X = S and Se, respectively. Every single layer is built from M^2+^ ions arranged in a honeycomb lattice, and every hexagon is centered by a P–P dimer perpendicular to the layer, forming [PX_3_]^2‐^ pyramids above and below the layer (**Figure** **2**A). Thus, every layer is terminated by chalcogen atoms, leading to weak van der Waals (vdW) interactions between layers. Following the crystallographic structure of MPX_3_, one can see that three types of chemical bonds—ionic, covalent, and vdW—are responsible for the formation of the electronic structure of these materials, challenging the correct description of their electronic properties.^[^ ^20^ ^]^ The representative high‐resolution transmission electron microscopy (TEM) images of several MPX_3_ materials are shown in **Figure** **3**.^[^ ^33^, ^34^, ^35^, ^36^ ^]^

All MPX_3_ materials are wide‐bandgap semiconductors. Among them, the MPX_3_ compounds consisting of open *d*‐shell M^2+^ ions are antiferromagnetically ordered, demonstrating different types of magnetic ordering; e.g., MnPS_3_ is a Heisenberg‐type Néel AFM with $T_{N}=87$ K, while FePS_3_ exhibits Ising‐type zigzag AFM ordering with $T_{N}=118$ K, etc.^[^ ^37^, ^38^, ^39^ ^]^ (Figure 2B). Along with that, TMTs that include two transition metal ions, such as CuCrP_2_S_6_, where the ionic charge states are Cr^3+^ and Cu^1+^, exhibit intralayer FM ordering of Cr magnetic moments and AFM coupling between neighboring layers.^[^ ^40^, ^41^ ^]^ The magnetic ordering of M^2+^ or M^3+^ ions in a single‐MPX_3_ layer is the result of the competition between exchange couplings among the first, second, and third nearest‐neighbor (NN, 2NN, and 3NN) localized spins, respectively. In turn, the NN exchange interaction $J_{1}$ arises from the competition between direct M–M exchange and M–X–M superexchange interactions (Figure 2B). The direct M–M exchange is always AFM, determined by the overlap of *d* orbitals, whereas the M–X–M superexchange interaction is always FM due to the M–X–M bond angle being close to 90°, following the well‐known Goodenough–Kanamori–Anderson rules^[^ ^42^, ^43^, ^44^ ^]^ (Figure 2B). Therefore, as a first perspective from this consideration, one can expect that the magnetic state of originally AFM‐ordered MPX_3_ compounds can be tuned and converted to a FM state if the M–X–M superexchange interaction begins to dominate over the direct M–M coupling. This can be realized in different ways, as will be discussed further.

The electronic structure of different MPX_3_ compounds has been rigorously studied in a series of recent experimental and theoretical works. Although several inconsistent and nonsystematic studies can be found in the literature (see discussion in ref. [20]), the current consensus is that most of these compounds are wide‐bandgap semiconductors and, in the case of magnetic M^2+^ ions, exhibit AFM ordering. The calculated and measured bandgaps for these materials vary between 1.2 and 3.5 eV, depending on the specific M and X elements (see examples of the calculated band structures in Figure 2C). Using various experimental techniques in combination with theoretical analysis, these materials have been classified into several types of insulating states according to the Zaanen–Sawatzky–Allen scheme^[^ ^45^ ^]^: MnPX_3_, FePX_3_, and CuCrP_2_S_6_ are described as Mott–Hubbard insulators ($\Delta >U_{dd}$)^[^ ^37^, ^41^, ^46^, ^47^, ^48^, ^49^, ^50^, ^51^, ^52^, ^53^ ^]^; NiPS_3_ is classified as a charge‐transfer insulator ($\Delta <U_{dd}$)^[^ ^46^, ^50^, ^54^ ^]^; and CoPS_3_ shows intermediate behavior ($\Delta \approx U_{dd}$).^[^ ^55^, ^56^ ^]^ Here, Δ denotes the charge‐transfer energy between the metal *d*‐states and ligand (chalcogen) *p*‐states, while $U_{dd}$ represents the on‐site Coulomb repulsion energy for the 3d states. Recent spectroscopic studies of these compounds have provided a more complete understanding of their electronic structure and have enabled detailed tracking of changes in the electronic properties across AFM phase transitions.

The most common method for synthesizing high‐quality MPX_3_ materials is chemical vapor transport (CVT).^[^ ^20^, ^21^, ^57^ ^]^ In this approach, the appropriate amounts of elements, in the correct molar ratio (M:P:X = 1:1:3), are mixed and sealed in an evacuated quartz ampule, with a small amount of $I_{2}$ typically added as a transport agent. The sealed ampule is then placed in a two‐zone furnace, where a specific temperature gradient and time are applied to produce high‐quality samples up to $1\;\;{\text{cm}}^{2}$ in size (see refs. [20, 21, 32]). However, the wide‐bandgap nature of these materials leads to some experimental challenges, e.g., in electron spectroscopy experiments, primarily due to potential charging effects in bulk or thick MPX_3_ samples (even during proper handling and mounting) or sample inhomogeneities that may occur during cleavage. These challenges can be addressed through various strategies, such as transferring very thin layers onto conductive supports; however, this introduces uncontrollable interfaces between the MPX_3_ layers and the substrate. A recently developed modified chemical vapor deposition technique for growing MPX_3_ layers on SiO_2_/Si substrates, as well as on fluorine‐doped tin oxide and indium tin oxide glass substrates, has shown promise in overcoming these issues.^[^ ^58^, ^59^ ^]^ Nevertheless, further experimental development is required for the large‐scale production of thin MPX_3_ layers, potentially up to several square millimeters.

## 3 Modifications of MPX_3_ Materials

This section provides an overview of the three main approaches currently proposed and employed for the functionalization of various vdW MPX_3_ materials, aiming to enhance their performance in different application areas. These approaches, along with corresponding references and brief results of the respective MPX_3_ modifications, are summarized in **Table** **1**, with emphasis on recent experimental and theoretical works.

**Table 1:** An overview of approaches to modifying the various MPX_3_ materials discussed in this review.

Modification	MPX_3_ material	Study approach	Result of modification	References
**Defects**				
X‐vacancy	MnPX_3_	Theory	Reduction of the bandgap; emergence of midgap states	[37, 76]
	NiPX_3_	Theory	Reduction of the bandgap; emergence of midgap states; formation of FM state	[60]
	Ni_1–*x*_Co_*x*_PS_3_	Experiment	Formation of FM state as a result of formation of S‐vacancies during samples’ synthesis	[66]
	CrPX_3_	Theory	Insignificant effect on electronic/magnetic properties	[61]
X/P‐vacancy	MnPX_3_ FePS_3_ NiPS_3_	Experiment	Prolonged electron beam irradiation leads to the gradual degradation and decomposition of MPX_3_ layers	[62, 63, 64]
	MnPS_3_	Experiment	Transformation into cubic *α*‐MnS alloyed with P	[63]
	MnPSe_3_	Experiment	Formation of mixed *α*‐MnSe and *γ*‐MnSe phases	[63]
	FePS_3_	Experiment/ theory	Reduction of the bandgap; emergence of midgap states; increased of water adsorption energy around defect places; desorption of S atoms and decomposition of FePS_3_ above $500^{\circ }\;C$ into amorphous phase (FeP/FeP_2_ alloyed with S)	[67]
	Co‐doped FePS_3_	Experiment/ theory	Increased surface conductivity of MPX_3_; increased stability towards electrocatalytic OER	[136]
M‐vacancy	MnPS_3_	Experiment	Weak ferromagnetism (Note: most probably due to the ion‐exchange mechanism during samples’ preparation)	[65]
**Adsorption**				
H_2_O	MnPX_3_	Theory	Water splitting is possible at defects; magnetic state is unchanged	[76]
	NiPX_3_	Theory	Molecular adsorption; magnetic state is unchanged	[60]
	CrPX_3_	Theory	Molecular adsorption; magnetic state is unchanged	[61]
	FePX_3_	Experiment/ theory	Increased of the water adsorption energy around defect places; low temperature and low partial pressure of H_2_: physisorption of H_2_O on FePX_3_; high temperature and high partial pressure of H_2_O: oxydation of the top FePX_3_ layer and formation of a P_*x*_O_*y*_ “dead” layer.	[67]
NH_3_, H_2_, CO, CO_2_, C_2_H_2_, H_2_S, CH_4_	MnPS_3_	Theory	Weak interaction; no significant effect on magnetic properties	[31, 80, 81]
NO_2_	MnPX_3_	Experiment	High sensitivity for NO_2_ adsorption	[78, 79]
NO_2_	MnPS_3_	Theory	High sensitivity for NO_2_ adsorption; strong adsorption of NO_2_ with formation of the P—O bond between molecule and MnPS_3_	[80]
NO_2_	MnPS_3_	Theory	Strong interaction; no significant effect on magnetic properties	[81]
NO	MnPS_3_	Theory	Strong interaction; substantial enhancement of the exchange interaction between Mn^2+^ ions; substantial increase in the magnetocrystalline anisotropy energy; induction of intralayer Dzyaloshinskii–Moriya interactions	[81]
CO_2_	FePS_3_	Experiment/ theory	Surprisingly high adsorption energy of CO_2_ on FePS_3_ (−2.2 eV) in the DFT calculations; spectroscopy and DFT studies on conversion of CO_2_ to CH_3_OH and C_2_H_5_OH	[70]
Li, F	MnPX_3_	Theory	Tuning the magnetic state of the top layers; emergence of a HMF state	[83, 84]
FM Co‐film	FePS_3_	Experiment	Magnetic exchange coupling at the interface; magnetic moments of Fe ions in FePS_3_ are aligned parallel to the magnetization of the Co thin film	[85]

**Intercalation**				
Li, Na, K	FePS_3_ CoPS_3_ NiPS_3_	Experiment	A redox‐type intercalation with reduction of the host lattice	[89, 97, 99, 100, 101, 102, 103]
	MnPS_3_	Experiment	A nonredox process with simple cation exchange without significant changes in the oxidation state of the transition metal	[89, 96, 97, 107, 108, 109, 110]
Li, Na	NiPS_3_	Experiment	Li intercalation (x<0.5 of Li per NiPS_4_ f.u.): an anionic redox mechanism in which Ni remains redox‐inactive; Li intercalation (x>0.5 of Li per NiPS_4_ f.u.): decomposition of NiPS_3_ with formation of Li_4_P_2_S_6_ and Li_2_	[106]
Li	NiPS_3_	Experiment	Electrochemical Li intercalation; ferrimagnetic state for intercalated compound at T≈2 K	[120]
Li, Na	HEA FeMnNiVZnPS_3_	Experiment/ theory	Aperiodic stacking in HEA and strain soliton boundaries promote the Na^+^‐ion diffusion	[33]
Li	FePS_3_	Theory	Emergence of a HMF state in intercalation compound	[137]
Li	FePS_3_	Experiment	Electrochemical Li intercalation; FM‐like state for intercalated compound with $T_{C}$ depending on Li concentration	[138]
Li(K) and NH_3_ co‐intercalation	FePS_3_	Experiment	Suppression of the AFM state in intercalation compounds; observation of spin‐glass state (at <50 K)	[139]
C_6_H_11_N_2_BF_4_ ionic liquid	FePS_3_ NiPS_3_	Experiment	Electrochemical interacalation of ionic liquid; no FM state; after intercalation: unaffected $T_{N}$ for NiPS_3_ and reduced $T_{N}$ for FePS_3_	[140]
[M(salen)]^+^, M = Mn^3+^, Fe^3+^, Co^3+^	MnPS_3_	Experiment	Ion‐exchange mechanism for intercalation; formation of Mn‐vacancies in intercalation compounds; observation of spontaneous magnetization for [Fe(salen)]^+^ and [Mn(salen)]^+^ with $T_{C}\approx 35$ K	[115]
TBA^+^‐cations	NiPS_3_	Experiment	Ferrimagnetic state for the intercalation compound with $T_{C}\approx 78$ K, hysteretic behavior with finite remanence and coercivity	[121]
THA^+^‐cations	NiPS_3_	Experiment/ theory	Ferrimagnetic state for the intercalation compound with $T_{C}\approx 100$ K, hysteretic behavior with large remanent magnetization and coercivity	[122]
Co(Cp)_2_^+^‐cations	NiPS_3_	Experiment	Ferrimagnetic state for the intercalation compound with $T_{C}\approx 98$ K, hysteretic behavior with finite remanence and coercivity	[121]
Co(Cp)_2_^+^‐cations	FePS_3_	Experiment	Ferrimagnetic state for the intercalation compound with $T_{C}<100$ K, hysteretic behavior with finite remanence and coercivity	[141]
PyH^+^‐cations	FePS_3_	Experiment	FM state for the intercalation compound with $T_{C}\approx 87$ K, hysteretic behavior with finite remanence and coercivity; out‐of‐plane magnetic anisotropy	[123]
TMA^+^‐, TEA^+^‐, TBA^+^, CTA^+^‐cations	MnPS_3_	Experiment/ theory	Nonredox ion‐exchange intercalation; formation of Mn‐vacancies in intercalation compounds; FM state for the intercalation compound with $T_{C}\approx 40-60$ K, hysteretic behavior with finite remanence and coercivity and large magnetic moment for Mn^2+^ ions	[124]

### 3.1 Defects Formation and Stability

**Figure 4:**
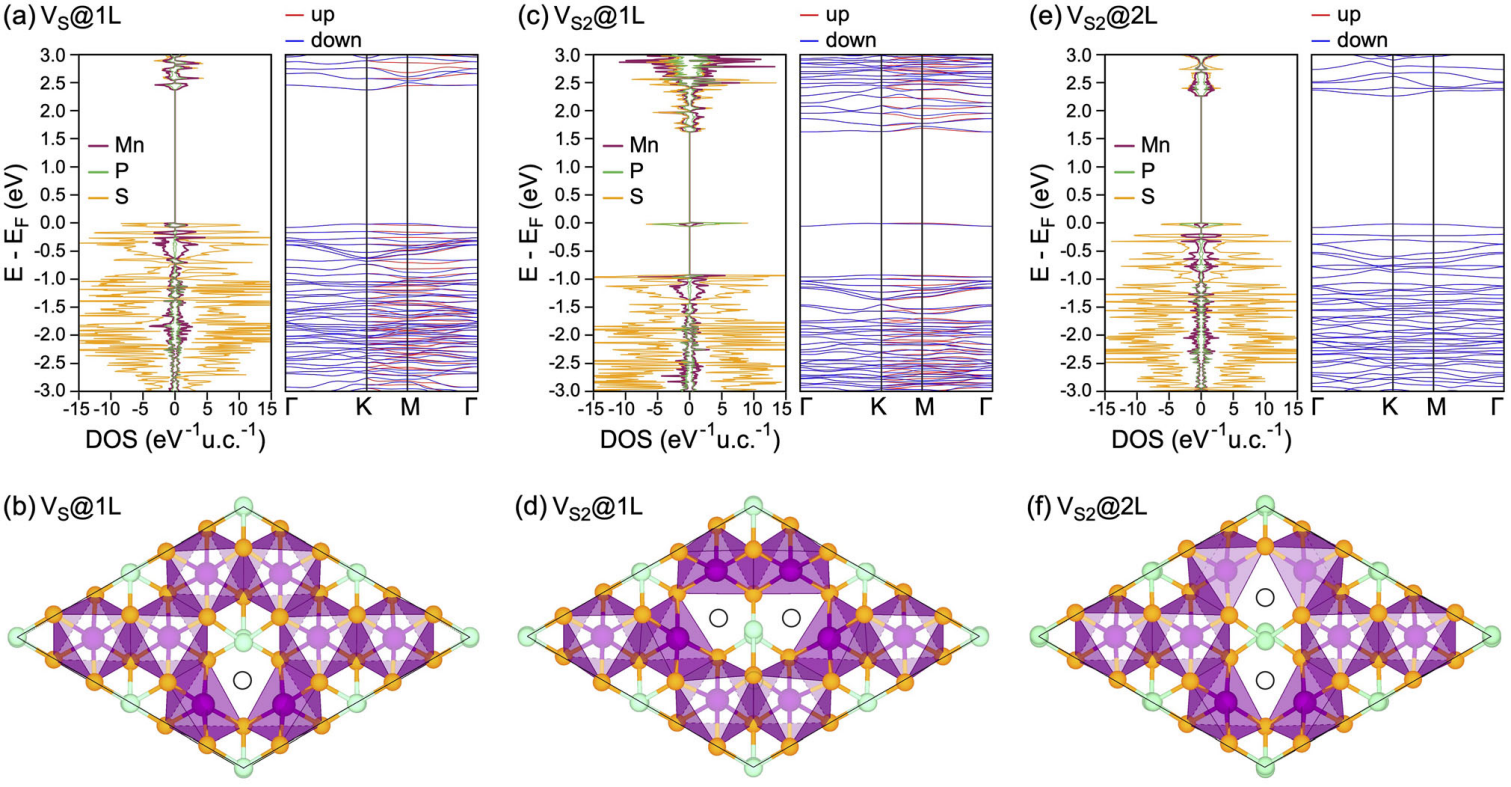
a,c,e) Atom‐projected DOS and band structures for defected MnPS_3_ single layers with single and double S‐vacancies with the corresponding structures presented in (b,d,f), respectively. Reproduced under the terms of the Creative Commons Attribution‐NonCommercial 3.0 Unported license.^[^ ^37^ ^]^ Copyright 2020, The Authors. Published by The Royal Society of Chemistry.

The creation of vacancies in MPX_3_ materials is a natural method for tailoring their electronic, magnetic, and optical properties. At the same time, it is expected that chalcogen or phosphorus vacancies may easily form during the exfoliation of MPX_3_ samples, potentially altering their properties and affecting the interpretation of observed phenomena. In a series of works,^[^ ^37^, ^60^, ^61^ ^]^ systematic density functional theory (DFT) calculations were performed for various chalcogen vacancy configurations in layers of MnPX_3_ (**Figure** **4**), NiPX_3_, and CrPX_3_. It was found that the formation energy for S and Se vacancies lies in the range of ≈1.2−1.6 eV, indicating a high likelihood of their formation during exfoliation. Furthermore, for the same defect type, Se vacancies are more likely to form than S vacancies, which correlates with their respective electronegativity values.

In the case of MnPX_3_ (Figure 4), it was found that if chalcogen vacancies appear within the same X‐layer, the resulting redistribution of electron density leads to a very weak ferrimagnetic state in MnPX_3_, with net total magnetic moments of ≈$0.001\;\mu_{B}$ (X: S) and $0.002\;\mu_{B}$ (X: Se).^[^ ^37^ ^]^ However, the formation of X‐vacancies in different chalcogen layers does not affect the AFM state of the MPX_3_ materials. The presence of such defects results in the emergence of midgap states in the case of double chalcogen vacancies within the same layer and leads to a reduction of the bandgap of MnPX_3_ in all studied cases. Surprisingly, no significant redistribution of electronic states in the valence or conduction bands of MnPX_3_ was observed, which leads to almost identical light absorption spectra for both pristine and defected MnPX_3_. Therefore, it was concluded that optical spectroscopy is not the most effective method for assessing the quality of MnPX_3_ materials and studied samples, and that further experimental techniques must be developed for this purpose.

The formation of X‐ and P‐vacancies in MnPX_3_, FePS_3_, and NiPS_3_ upon irradiation with a high‐energy electron beam in TEM experiments was observed in refs. [62, 63, 64]. Corresponding simulations of the electron beam interactions with these materials showed that X‐vacancies form at significantly lower acceleration voltages compared to P‐vacancies—≈60 keV versus 95 keV, respectively—an effect that is clearly related to the crystallographic structure of MPX_3_ layers. Notably, both of these energy thresholds are considerably lower than those required to create vacancy defects in graphene, which is a critical factor in the context of TEM investigations. It should also be noted that prolonged electron beam irradiation leads to the gradual degradation and decomposition of MPX_3_ layers. Extended exposure results in a change in the sulfur concentration within the layer, followed by oxidation driven by residual gases present in the microscope environment.^[^ ^62^ ^]^

It is interesting to consider the observation of the so‐called “parasitic” ferromagnetism in few‐layered MnPS_3_ prepared via liquid exfoliation,^[^ ^65^ ^]^ which was also attributed to the formation of vacancies in this material. The MnPS_3_ samples were produced by stirring CVT‐grown crystals in a KCl solution, followed by centrifugation and decantation. The resulting few‐layered MnPS_3_ exhibited very weak FM ordering below a transition temperature of 38 K, which was explained by the formation of Mn‐vacancies introduced during the exfoliation process. However, considering the crystallographic structure of MnPS_3_, the formation of vacancies within the central Mn layer of the lattice without causing structural disintegration of the entire crystal appears unlikely. Instead, the formation of S‐ and P‐vacancies during synthesis is more plausible. Nevertheless, as was demonstrated theoretically^[^ ^37^ ^]^ and discussed earlier, such vacancies in MnPS_3_ are not expected to generate a significant magnetic moment. The most probable mechanism for the emergence of weak ferromagnetism in MnPS_3_ is the intercalation of Li ions between the layers during the sample preparation process described above, which can lead to the ion‐exchange process with formation of Mn vacancies in the MnPS_3_ layers. This interpretation is supported by the observed increase in interlayer spacing—from 6.51 Å in bulk MnPS_3_ to 7.55 Å in the few‐layered samples.^[^ ^65^ ^]^ As will be discussed in more detail in Section 3.2 and 3.3, Li intercalation can also lead to the effective doping the MnPS_3_ layers, leading to an enhancement of the Mn–S–Mn FM superexchange interaction for the $J_{1}$ NN coupling. This enhancement can overcome the intrinsic AFM direct exchange, resulting in the observed weak ferromagnetism. Further details of possible mechanism are presented below.

**Figure 5:**
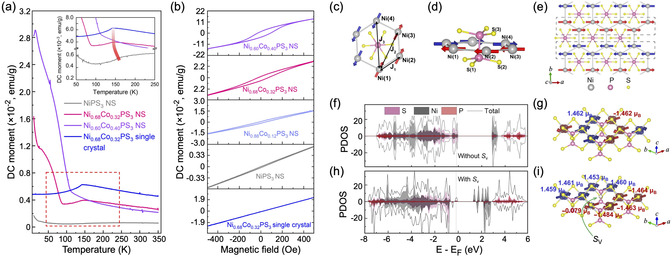
a) Temperature dependence of the zero‐field cooled (ZFC) magnetization measured at H=200 Oe on various Ni_1–*x*_Co_*x*_PS_3_ nanosheets (x=0,0.32,0.40) and the Ni_0.68_Co_0.32_PS_3_ single crystal. b) M−H curves measured at 5 K of various Ni_1–*x*_Co_*x*_PS_3_ nanosheets (x=0,0.12,0.32,0.40) in comparison with that of the Ni_0.68_Co_0.32_PS_3_ single crystal. c,d) The illustrations of the exchange interactions in a hexagonally arranged lattice of Ni neighbors in NiPS_3_ mediated via S atoms. e) Top view of a NiPS_3_ ML with alternating FM chains marked with dashed black boxes, coupled antiferromagnetically throughout the crystal lattice. f,h) Spin‐polarized atom‐projected DOS and g,i) the corresponding illustrations of the magnetic moment distribution of AFM NiPS_3_ without S vacancy and with S vacancy. Reproduced under the terms of the Creative Commons Attribution NonCommercial license 4.0 (CC BY‐NC).^[^ ^66^ ^]^ Copyright 2021, The Authors. Published by The American Association for the Advancement of Science.

The interesting situation was found for defects formation in layers of NiPX_3_.^[^ ^60^ ^]^ In this systematic DFT calculations, the formation of the double‐X vacancies in NiPX_3_ is found to be more energetically favorable compared to other types. In this case, the removal of a chalcogen atom breaks the bond, and the remaining electron occupies the easily available electronic states of a (P_2_X_5_) entity. As a result, a localized defect state appears in the energy gap just at the top of the valence band, and the electron density is delocalized between the P and X atoms of the defective sublayer. Also, one more state is formed in the energy gap between the valence and conduction bands, and it has Ni 3d character. The magnetic moments of the Ni^2+^ ions near the vacancy are coupled ferromagnetically, and both considered states appear in the spin‐up channel. Naturally, these changes in the electronic structures of both materials lead to the decrease of the energy gap width to 1.36 eV/1.29 eV for the defective NiPS_3_/NiPSe_3_, compared to 2.19 eV/1.85 eV for pristine NiPS_3_/NiPSe_3_, respectively. These theoretical findings were confirmed by the experimental observations of FM order in Ni_1–*x*_Co_*x*_PS_3_ caused by the formation of S‐vacancies^[^ ^66^ ^]^ (**Figure** **5**). Here, Ni_1–*x*_Co_*x*_PS_3_ nanosheets (NS) were synthesized via the chemical vapor conversion method, and it was demonstrated that a weak FM ground state in these compounds is formed at low temperatures, with a transition temperature depending on *x*: it gradually increases from 25 to 110 K, when *x* is changed from 0 to 0.4. The respective theoretical analysis shows that the presence of S‐vacancies in Ni_1–*x*_Co_*x*_PS_3_ is responsible for the suppression of long‐range AFM order, while other competing FM interactions start to dominate at low temperatures. The corresponding DFT calculations show that the charge redistribution around S‐vacancies leads to an uncompensated magnetic moment of $\approx 0.17\;\mu_{B}$/f.u. In this case, the imbalance between spin‐up and spin‐down electrons in two antiferromagnetically coupled sublattices leads to the prevailing of FM interaction over the AFM correlations at low temperatures.

**Figure 6:**
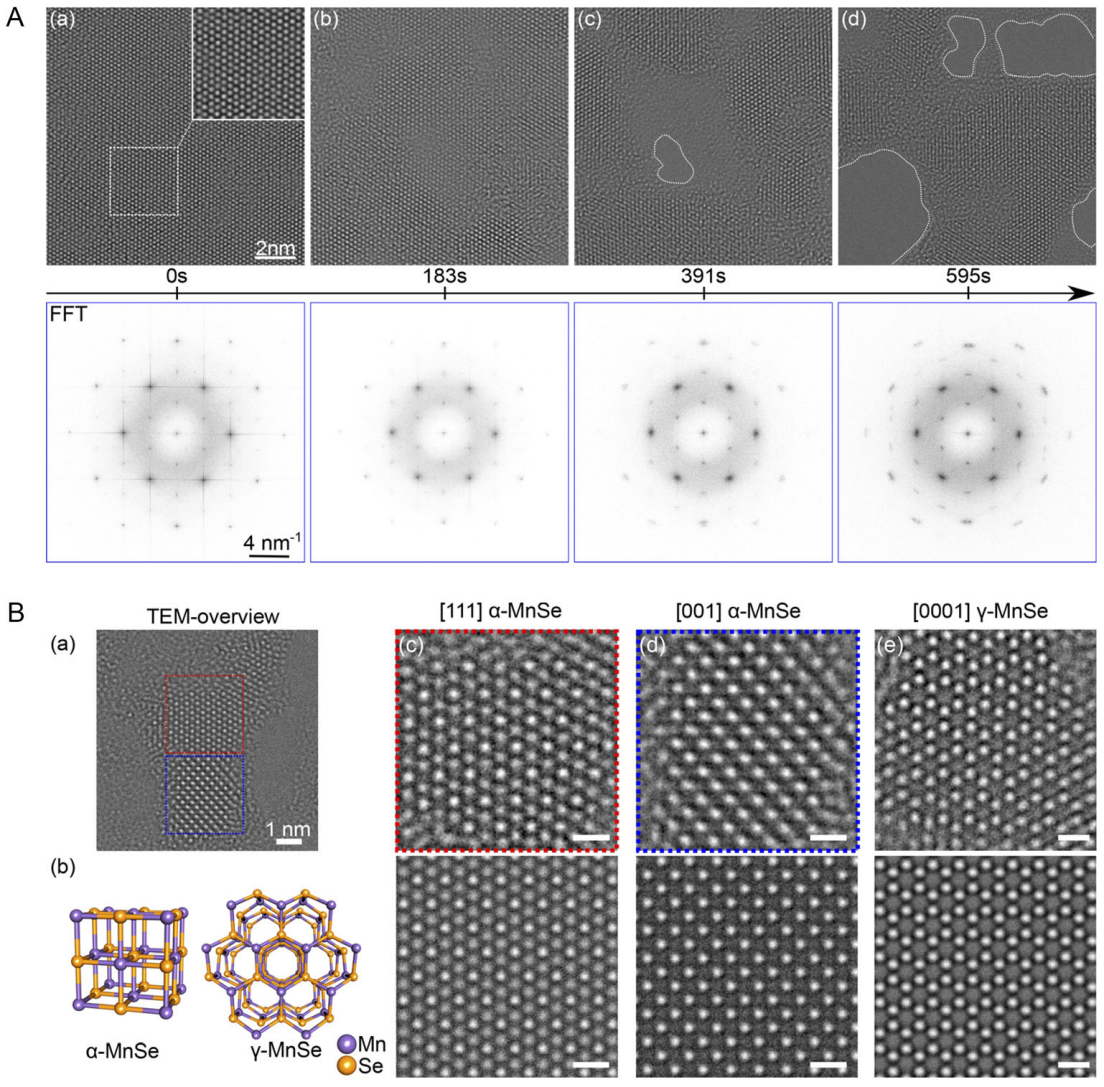
A): a–d) 80 kV Cc/Cs‐corrected high‐resolution TEM (HRTEM) image series of few‐layer MnPSe_3_ acquired with a dose rate of $0.6\times 10^{6}\frac{e}{nm^{2}}$, illustrating the degradation process under electron beam irradiation. Underneath each HRTEM image, the corresponding FFT image is shown. Damage caused by the e‐beam irradiation can be observed in the images, and the formation of holes is indicated by the white dotted lines. B): a) TEM overview image of an irradiated MnPSe_3_ crystal proofing the emergence of new phases highlighted by dotted boxes; b) model of the emerging phases, namely, of cubic *α*‐MnSe ($Fm\bar{3}m$) and hexagonal *γ*‐MnSe ($P6_{3}mc$); c–e) HRTEM images of experimentally observed MnSe patches in different orientations and types. Corresponding simulations are given beneath the experimental images. The scale bars in image panels (c–e) represent a length of 0.5 nm. Reproduced with permission.^[^ ^64^ ^]^ Copyright 2023, American Chemical Society.

**Figure 7:**
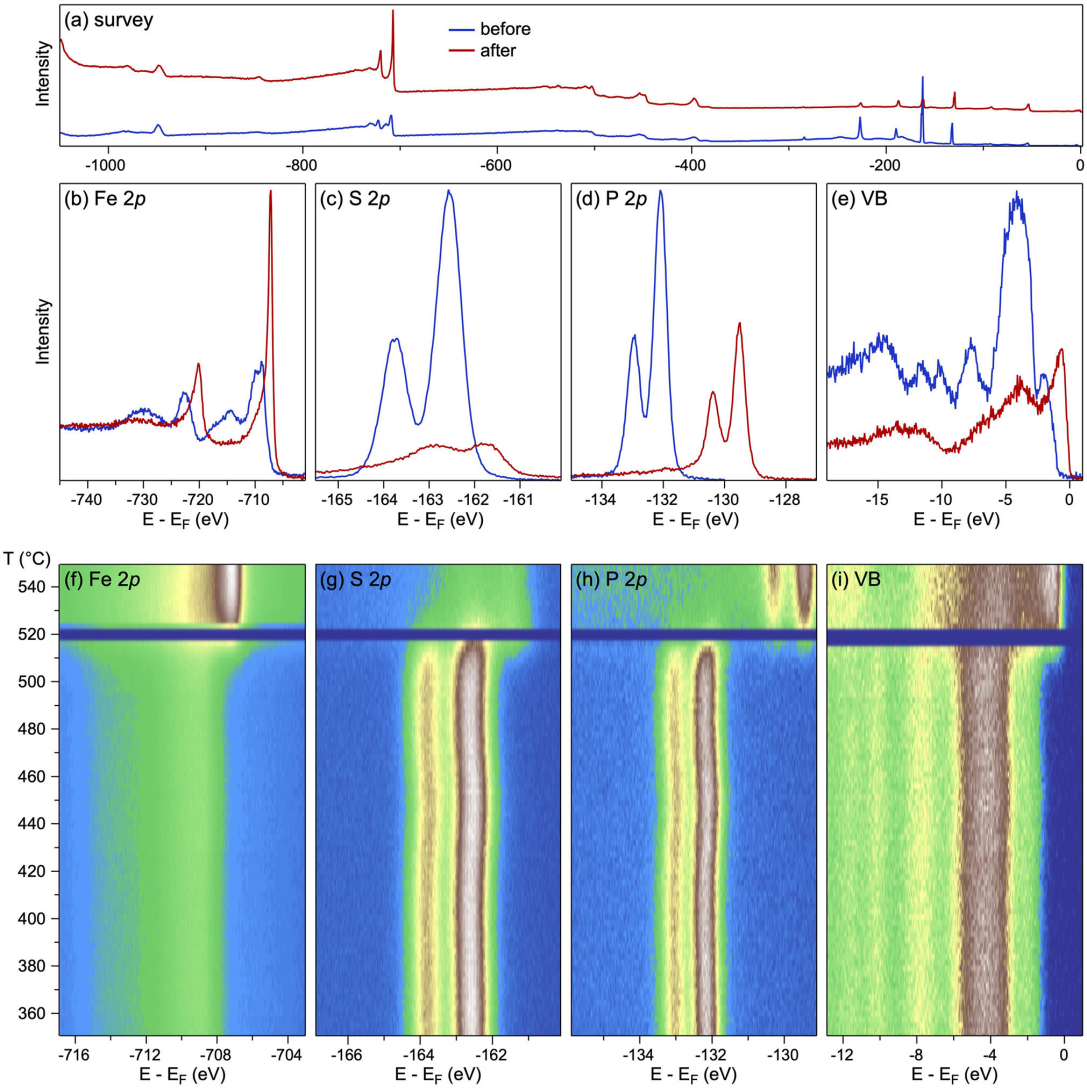
XPS spectra (measured at room temperature) collected for FePS_3_ before and after temperature stability experiments: a) survey, b) Fe 2p, c) S 2p, d) P 2p, and e) valence band. f–i) Photoemission intensity maps based on the sequences of the respective XPS spectra collected in the “snapshot” mode of the analyzer as a function of the sample temperature. Spectra are shown in the temperature range of $350-550^{\circ }C$. The temperature gradient is 3 degrees per minute. All spectra were collected at photon energy of hν=1000 eV. Horizontal intensity drops in (f–i) are due to the closed last valve of the beamline when pressure in the chamber raised above the working limit. Reproduced with permission.^[^ ^67^ ^]^ Copyright 2023, IOP Publishing.

As discussed earlier, the irradiation of MPX_3_ materials with a high‐energy electron beam can lead to changes in the materials’ stoichiometry and, as later found, to the restructuring of trichalcogenides into new materials with different crystallographic structures. Such experiments show that in the case of MnPS_3_, the electron beam irradiation leads to a transformation into cubic *α*‐MnS alloyed with P,^[^ ^63^ ^]^ whereas for MnPSe_3_, the formation of mixed cubic *α*‐MnSe and hexagonal *γ*‐MnSe phases is observed^[^ ^64^ ^]^ (**Figure** **6**). Additionally, the thermal stability experiments for these materials show that both MnPX_3_ materials are converted under vacuum conditions upon thermal annealing into cubic *α*‐MnS/MnSe phases at 490°C/400°C, respectively. Similar thermal stability experiments performed on FePS_3_ showed different results^[^ ^67^ ^]^ (**Figure** **7**). In this case, bulk FePS_3_ crystals were annealed in ultrahigh vacuum conditions, and the so‐called “live” X‐ray photoelectron spectra (XPS) of the respective core levels were acquired. In contrast to MnPX_3_, in the case of FePS_3_, the predominant desorption of S atoms is observed, and above 500 °C, this material decomposes into a new amorphous phase consisting of iron phosphides FeP/FeP_2_ alloyed with S, as demonstrated by XPS data.^[^ ^67^ ^]^

### 3.2 Adsorption of Different Species

With the recent progress in the studies of 2D and vdW materials, MPX_3_ materials, as wide‐bandgap materials, have attracted much attention due to the possibility of using them in water‐splitting reactions for hydrogen evolution and oxygen evolution reactions (HER and OER),^[^ ^68^, ^69^ ^]^ as well as for CO_2_ gas conversion into methanol and/or ethanol.^[^ ^70^ ^]^ It has been shown that most of these materials ideally combine the width of the bandgap and the positions of the bands’ edges with respect to redox potentials at different pH values, along with relatively high mobility for electrons and holes.^[^ ^71^ ^]^ Many experimental and theoretical works have been devoted to these studies,^[^ ^72^, ^73^, ^74^, ^75^ ^]^ yet a systematic approach for considering the adsorption of different species on the surface of MPX_3_ has been lacking, raising many controversies and critical questions regarding the interpretation of structural, spectroscopic, and electrochemical data. Thus, later, a series of works were dedicated to analyzing water adsorption on both pristine and defective MPX_3_, with a deeper understanding of the possible mechanisms that might govern the effective HER and OER on these materials.

**Figure 8:**
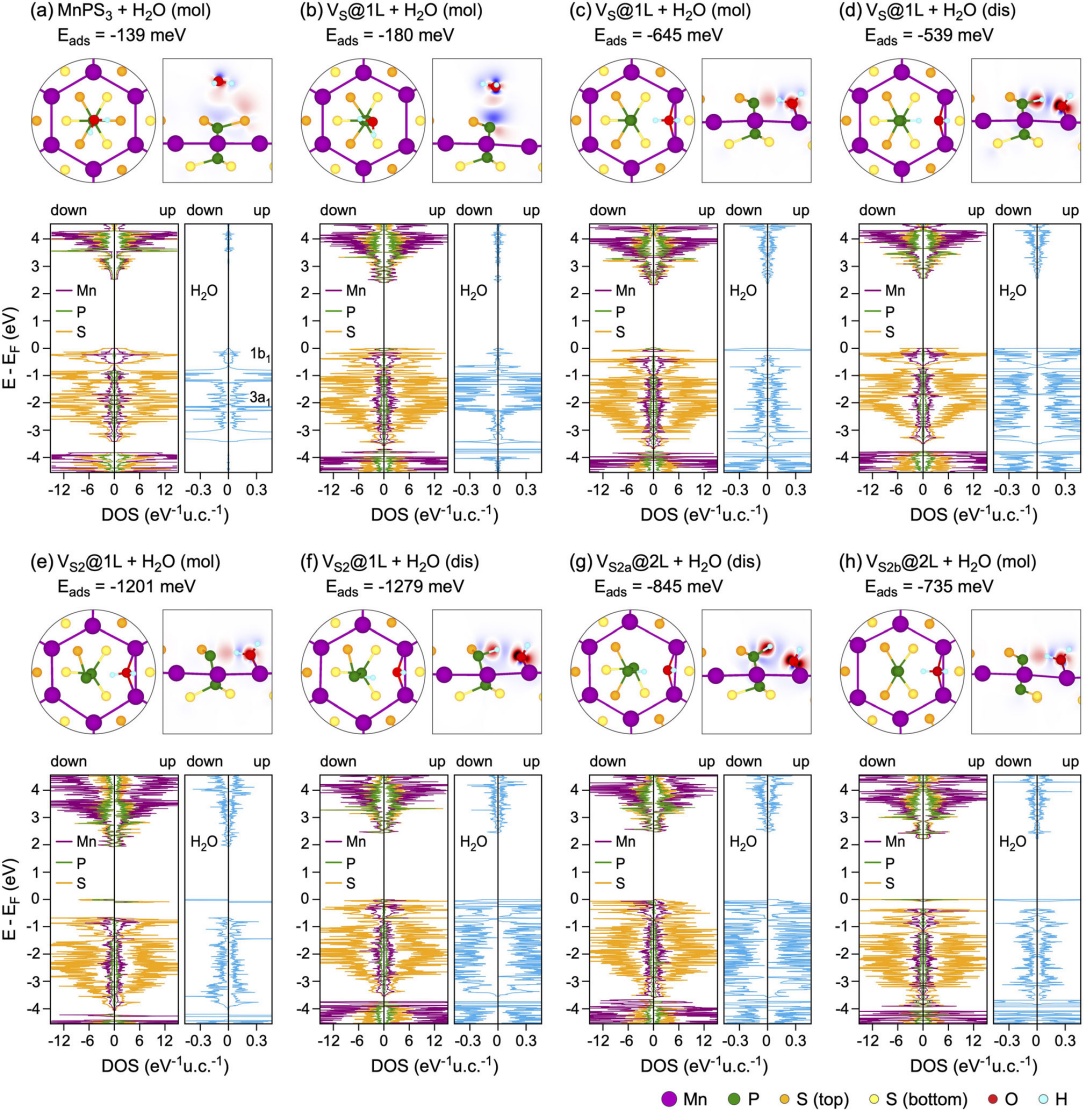
Top and side views of the relaxed structures obtained after water adsorption on pristine and defective MnPS_3_. Spheres of different size/color represent ions of different types. Side views are superimposed with electron density redistribution maps: $\Delta \rho (r)=\rho_{A/B}(r)-[\rho_{A}(r)+\rho_{B}(r)]$ (A: adsorbate; B: substrate). Here, Δρ(r) is color‐coded as blue (−0.01 eÅ^−3^)—white (0)—red (+0.01 eÅ^−3^) in (a,b) and as blue (−0.1 eÅ^−3^)—white (0)—red (+0.1 eÅ^−3^) in (c–h). Bottom part of each panel presents the atom‐projected DOS obtained with PBE + *U* + D2 for the respective structures. Reproduced with permission.^[^ ^76^ ^]^ Copyright 2023, American Chemical Society.

The adsorption of H_2_O molecules on the surface of pristine MPX_3_ materials is always a physisorption process, with the adsorption energy not exceeding −210 meV per molecule.^[^ ^60^, ^61^, ^67^, ^76^ ^]^ Only the formation of chalcogen vacancies, which are the most probable defects in the MPX_3_ layer, leads to a drastic increase in adsorption energy. For example, the adsorption energy is −572 meV per H_2_O molecule for a single S‐vacancy in NiPS_3_^[^ ^60^ ^]^ and −1279 meV per H_2_O molecule for a double S‐vacancy in MnPS_3_^[^ ^76^ ^]^ (**Figure** **8**). The dissociative adsorption of H_2_O, when it is split into H^+^ and OH^−^ fragments, is not always energetically favorable, demonstrating either positive adsorption energies (as observed for ideal NiPX_3_ and MnPX_3_ surfaces^[^ ^60^, ^76^ ^]^) or yielding adsorption energies smaller than those for nondissociative water adsorption (as observed for defective NiPX_3_ and CrPX_3_^[^ ^60^, ^61^ ^]^). In the case of MnPX_3_, it has been found that for all X‐defective MnPX_3_ surfaces, the adsorption energy for H_2_O molecules is higher compared to the pristine surface.^[^ ^76^ ^]^ Additionally, the energies for dissociative adsorption of H_2_O on the defective MnPX_3_ surfaces become comparable with those for nondissociative adsorption, demonstrating the possibility of implementing HER on this material. In the same study,^[^ ^76^ ^]^ the Gibbs free energies of the intermediate state, $\left|\Delta G_{H^{*}}\right|$, for different MnPX_3_ surfaces (both pristine and defective) were calculated and compared with $|\Delta G_{H^{*}}|=0.09$ eV for the well‐known highly efficient Pt catalyst. Among all possible configurations, the surface with double X‐vacancies demonstrated the lowest values of $|\Delta G_{H^{*}}|=0.69\;\text{eV}/0.41\;\text{eV}$ for X = S/Se,^[^ ^76^ ^]^ respectively, which are comparable with the corresponding experimental values found in the range between 0.525 and 0.835 eV.^[^ ^21^, ^77^ ^]^

A similar behavior is observed for FePX_3_ materials, where the creation of single X‐vacancies in the top FePX_3_ layer leads to an increase in the adsorption energy of water to −712/−715 meV per H_2_O molecule, compared to −209/−207 meV for X = S/Se, respectively.^[^ ^67^ ^]^ This molecular adsorption is slightly more energetically favorable than the dissociative adsorption of water molecules on defective FePSe_3–*x*_, which exhibits a slightly lower adsorption energy of −649 meV per H_2_O molecule. These theoretical predictions are supported by corresponding XPS and NEXAFS (near‐edge X‐ray absorption fine structure) studies on water adsorption on FePX_3_ under ultrahigh vacuum conditions, confirming the physisorption nature of the H_2_O–FePX_3_ interaction in the temperature range of 100−300 K and under low H_2_O partial pressures.^[^ ^67^ ^]^ Additional XPS/NEXAFS measurements at elevated temperatures (above 573 K) and higher water partial pressures (above 0.1 mbar) reveal two competing processes: adsorption at high H_2_O partial pressure and desorption due to increased surface mobility and the physisorptive nature of the interaction. Further heating of FePX_3_ to 673 K results in partial oxidation of the top layer, leading to the formation of a P_*x*_O_*y*_ “dead” layer.

The experimentally observed high gas selectivity of MnPX_3_ towards NO_2_ under ambient conditions^[^ ^78^, ^79^ ^]^ was recently analyzed using DFT calculations.^[^ ^80^ ^]^ Theoretical results show that the adsorption energy for various molecules (NH_3_, H_2_, CO, CO_2_, C_2_H_2_, H_2_S, CH_4_) does not exceed −160 meV per adsorbed molecule. In contrast, NO_2_ molecules adsorb on MnPS_3_ with a much higher energy of −640 meV per NO_2_ molecule. Upon adsorption, one of the N—O bonds in NO_2_ breaks, and a P—O bond forms between NO_2_ and MnPS_3_. Furthermore, a significant increase in the density of states (DOS) occurs near the Fermi level, resulting in a pronounced change in the electrical conductivity of MnPS_3_ and a high response to NO_2_ molecules.^[^ ^80^ ^]^

As discussed in Section 2, the NN magnetic coupling in MPX_3_ materials can be tuned through various mechanisms. One particularly promising approach involves enhancing the M–X–M superexchange interaction to surpass the direct M–M exchange in determining the $J_{1}$ coupling parameter. The influence of molecular adsorption on the magnetic properties of MnPS_3_ was investigated in ref. [81], focusing on several species including CO, N_2_, NH_3_, NO, and NO_2_. In all cases, adsorption was characterized as physisorption, with adsorption energies ranging from ≈−100 to −200 meV per molecule for CO, N_2_, and NH_3_, which are similar to previous works. In contrast, significantly stronger adsorption was observed for NO and NO_2_, with energies of −446 and −492 meV, respectively. These values are lower by ≈150 meV compared to those previously reported in ref. [80] for the adsorption of NO and NO_2_, which can be attributed to slightly different computational settings used in these highly sensitive calculations. Further analysis revealed that NO molecules induce the most pronounced changes in the magnetic properties of the MnPS_3_ layer, resulting in a substantial enhancement of the exchange interaction between Mn^2+^ ions. This effect is attributed to the emergence of a new exchange pathway mediated by the NO molecules, which surpasses the conventional Mn–S–Mn superexchange interaction. Additionally, NO adsorption leads to a substantial increase in the magnetocrystalline anisotropy energy and induces intralayer Dzyaloshinskii–Moriya interactions. As was shown, these effects are likely a consequence of lattice distortions in MnPS_3_ following NO adsorption.

A more effective strategy for modifying the magnetic properties of MPX_3_ through adsorption is inspired by studies on doping MnPSe_3_ layers using an external electric field.^[^ ^82^ ^]^ It was demonstrated that both electron and hole doping can be experimentally realized at feasible carrier concentrations, leading to the emergence of a half‐metallic ferromagnetic (HMF) state.

**Figure 9:**
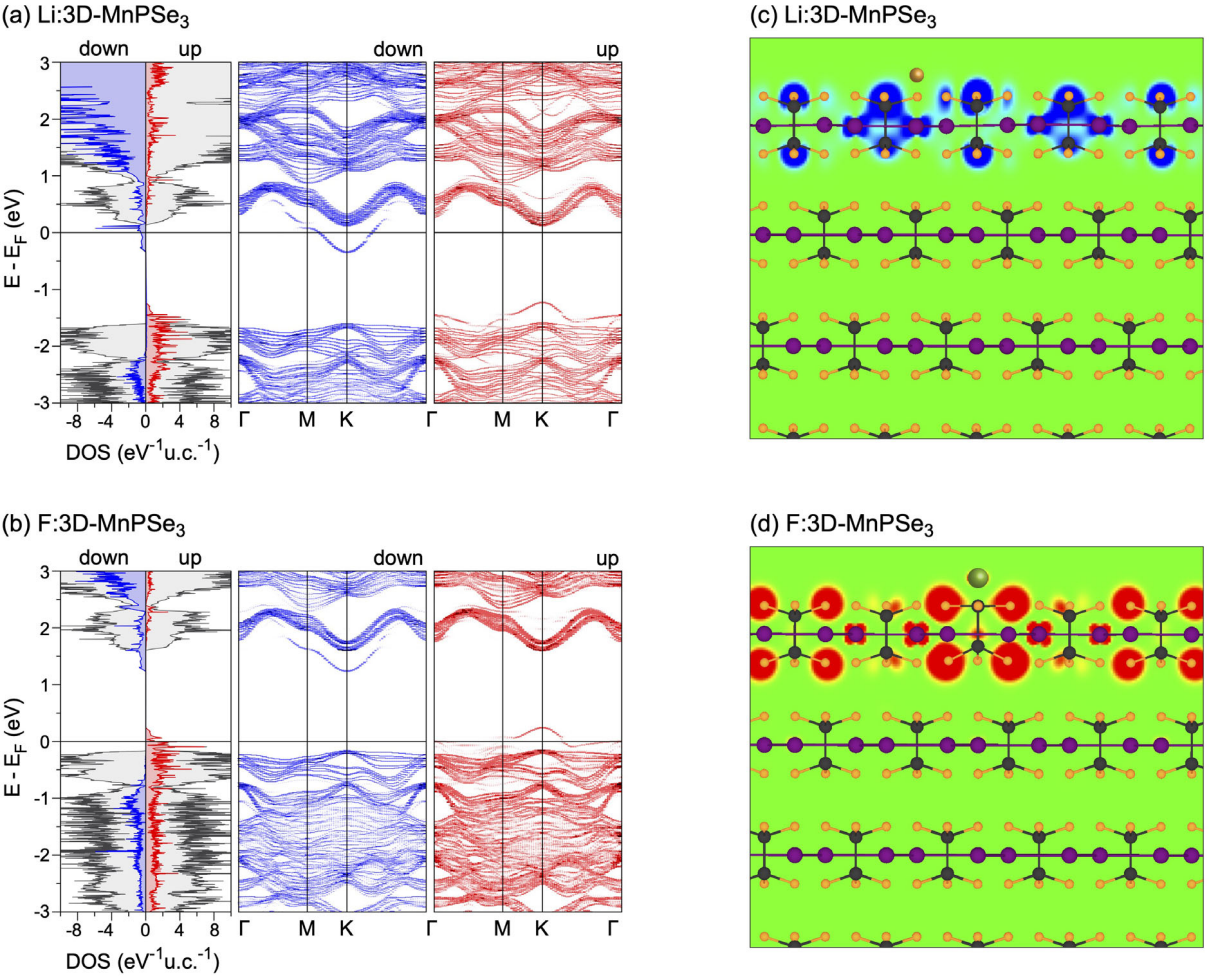
Spin‐resolved DOS and band structures for 3D MnPSe_3_ after adsorption of Li a) (Li:3D‐MnPSe_3_) and F b) (F:3D‐MnPSe_3_). In DOS plots, the grey shaded areas indicate the total DOS and the blue/red shaded areas indicate the spin‐down/spin‐up DOS projected onto the atoms of the outermost MnPSe_3_ layers. c,d) The real‐space spin‐density distribution maps overlaid with the respective structures for Li:3D‐MnPSe_3_ and F:3D‐MnPSe_3_, respectively. The maps are color‐coded as blue (−0.001 eÅ^−3^), green (0), and red (+0.001 eÅ^−3^). Reproduced with permission.^[^ ^83^ ^]^ Copyright 2022, The Royal Society of Chemistry.

Building on the concept of doping or charge transfer in MPX_3_‐based systems, a study investigating the adsorption of Li and F atoms on stacked MnPX_3_ layers demonstrated that a HMF state can be realized selectively in the topmost MnPX_3_ layer, while the underlying bulk‐like layers remain largely unaffected^[^ ^83^ ^]^ (**Figure** **9**). Remarkably, tuning the magnetic state of the top layers of both MnPX_3_ compounds can be achieved with as little as 0.25 monolayers (MLs) of adsorbed guest atoms. Specifically, MnPS_3_ transitions to an HMF state upon Li adsorption, but retains its AFM character in the presence of F. In contrast, MnPSe_3_ exhibits an HMF state upon adsorption of either Li or F. It was found that Li atoms preferentially adsorb above Mn sites, while F atoms favor adsorption above P atoms. In all cases, charge redistribution occurs in the top MnPX_3_ layer: Li adsorption leads to charge accumulation on Mn—S/Se bonds, whereas F adsorption results in charge accumulation primarily on S or Se atoms. This redistribution modifies the magnetic exchange interactions, particularly enhancing the superexchange Mn–S–Mn interaction upon Li adsorption, thereby increasing the magnitude and altering the sign of all *J* values. As a result, the HMF state emerges, with Curie temperatures of $T_{C}=198$ and 126 K for MnPS_3_ and MnPSe_3_, respectively. F adsorption is less effective, yielding an HMF state only in MnPSe_3_, where the NN exchange ($J_{1}$) becomes negligible and the magnetic coupling is dominated by the 3NN interaction ($J_{3}$), leading to a Curie temperature of $T_{C}=85$ K. Due to the vdW nature of interlayer interactions in MnPX_3_, even with substantial electron or hole doping of the topmost layer, the inner layers remain in an AFM state with a wide bandgap. As proposed in ref. [83], such a configuration could be harnessed to construct an electric‐field‐driven “one‐material”‐based magnetic tunnel junction. This setup would feature ideal interfaces between conductive and insulating layers, minimizing electron scattering and improving performance in potential spintronic devices. These theoretical predictions were later supported in ref. [84], where it was also suggested that an HMF state in MnPS_3_ can be induced by adsorption on a metallic substrate such as Au(111). This phenomenon obtained theoretically for MnPS_3_ may be taken as another mechanism for the previously experimentally observed “parasitic” ferromagnetism in few‐layer MnPS_3_.^[^ ^65^ ^]^

A more straightforward approach to modifying the magnetic state of MPX_3_ materials involves the adsorption of thin ferromagnetic layers. However, only a limited number of studies on this topic are available in the literature. In ref. [85], a relatively thick Co film (≈7 nm) was deposited onto FePS_3_, and the magnetic properties of the resulting interface were investigated using magneto‐optical Kerr effect microscopy and X‐ray magnetic circular dichroism (XMCD). The Co film exhibited isotropic magnetic behavior, suggesting the absence of a preferred magnetic anisotropy direction. This was attributed to the lack of layer‐by‐layer growth during deposition, likely caused by the significant surface energy mismatch between Co and FePS_3_. Low‐temperature magnetic measurements revealed no clear exchange bias in the FM‐Co/AFM‐FePS_3_ system. However, a sharp reduction in coercivity was observed above 110−120 K, near the Néel temperature of FePS_3_, indicating the presence of exchange coupling at the interface. Unfortunately, no low‐temperature XMCD data were provided for the Co/FePS_3_ system, and only room‐temperature spectroscopic measurements, conducted well above the $T_{N}$ of FePS_3_, were reported. From the NEXAFS and XMCD spectra at the Fe $L_{2,3}$ absorption edges, a mixed Fe^2+^/Fe^3+^ signal was detected in the Co/FePS_3_ sample, in contrast to the purely Fe^2+^ signal observed in pristine FePS_3_.^[^ ^49^ ^]^ Control experiments confirmed that the appearance of Fe^3+^ is linked to the formation of the Co/FePS_3_ interface, corroborating previous observations in Co‐ and Ni‐doped FePS_3_ systems.^[^ ^86^, ^87^ ^]^ These results also indicate that magnetic exchange coupling occurs at the interface between FM Co and paramagnetic (at room temperature) FePS_3_, as evidenced by the dichroic signal observed at the Fe $L_{2,3}$ edges. The magnetic moments of Fe ions were found to align parallel to the magnetization of the Co thin film. Nevertheless, further experimental and theoretical investigations are required to elucidate the nature of this magnetic proximity effect, including the observed reorientation of FePS_3_ magnetic moments from out‐of‐plane to in‐plane alignment.

### 3.3 Intercalation of Different Species

The discussed MPX_3_ materials, as representative examples of layered compounds, are well suited for modification via intercalation—a widely used technique in which guest species are inserted into the available voids of the host material. In the case of MPX_3_, a natural intercalation pathway involves the insertion of guest atoms or molecules into the interlayer regions, which are initially held together by weak vdW forces. This approach enables significant tuning of the material's electronic and magnetic properties without disrupting the in‐plane atomic structure.

As previously discussed in the literature,^[^ ^88^, ^89^ ^]^ several well‐established methods exist for the intercalation of layered materials. These include direct thermal reactions between the host lattice and guest species, indirect reactions involving auxiliary processes that provide a net gain in free enthalpy, electrochemical intercalation using an appropriate electrolytic cell with the host lattice as the cathode, and ion‐exchange reactions. In general, intercalation leads to charge transfer between the guest species and the host lattice, which can profoundly affect the electronic, optical, and magnetic properties of the parent compound. Additionally, intercalation typically results in an expansion of the interlayer spacing (i.e., an increase in the lattice parameter *c*). These structural and electronic changes may also give rise to further modifications, such as alterations in stacking order, increased structural disorder, and the formation of distinct intercalation stages. During the 1970s–1990s, numerous experimental studies explored the intercalation of MPX_3_ materials with a wide range of guest species—from small alkali metals like lithium to larger organic molecules such as cobaltocene (Co(C_5_H_5_)_2_) and pyridine.^[^ ^57^, ^90^, ^91^, ^92^, ^93^ ^]^ In the present work, we focus on more recent efforts aimed at achieving two primary objectives through intercalation in MPX_3_ systems: 1) their application as electrode materials in rechargeable batteries and 2) the tuning of their magnetic ordering.

Extensive research has been devoted to understanding the mechanism of lithium intercalation in various MPX_3_ compounds.^[^ ^89^, ^94^, ^95^, ^96^ ^]^ It was found that the reaction of these materials with *n*‐butyl‐lithium, commonly used as the main agent in electrochemical intercalation, proceeds differently depending on the specific transition metal involved. These differences have been attributed to the electronic configuration of the M^2+^ ions, particularly the occupancy of their 3d orbitals. For instance, FePS_3_, CoPS_3_, and NiPS_3_, which have partially filled 3d shells, undergo rapid intercalation with *n*‐butyl‐lithium. In contrast, MnPS_3_, with a half‐filled $3d^{5}$ configuration, requires significantly longer reaction times—often several weeks—to achieve noticeable Li intercalation.^[^ ^89^, ^97^ ^]^ Based on these findings, two distinct intercalation mechanisms have been proposed for MPX_3_ compounds: 1) a redox‐type intercalation, involving reduction of the host lattice (as observed in FePS_3_, CoPS_3_, and NiPS_3_) and 2) a nonredox process, characterized by simple cation exchange without significant changes in the oxidation state of the transition metal (as in the case of MnPS_3_ and CdPS_3_).^[^ ^89^, ^96^, ^98^ ^]^

The reduction of the host lattice during lithium intercalation in NiPS_3_ has been investigated in several studies using various spectroscopic techniques. Early evidence was provided by nuclear magnetic resonance (NMR) and Mössbauer spectroscopy,^[^ ^99^, ^100^ ^]^ and was later revisited using Raman spectroscopy and extended X‐ray absorption fine structure analysis.^[^ ^101^, ^102^, ^103^ ^]^ More direct confirmation of the reduction of Ni^2+^ ions to metallic Ni^0^ upon Li intercalation was obtained through XPS.^[^ ^96^ ^]^ A comparison of the XPS spectra for pristine NiPS_3_ and Li_*x*_NiPS_3_ reveals notable shifts in the core‐level binding energies: the Ni 2p and 3p peaks shift to lower binding energies in the intercalated compound, while the P 2p peak shifts to higher binding energy. After intercalation, the Ni emission lines approach those characteristic of metallic nickel, and the satellite features typically present in the Ni 2p spectrum of Ni^2+^ (refs. [104, 105]) are significantly suppressed. These spectral changes provide compelling evidence for the reduction of Ni^2+^ ions to Ni^0^, strongly supporting the redox intercalation model in NiPS_3_.

**Figure 10:**
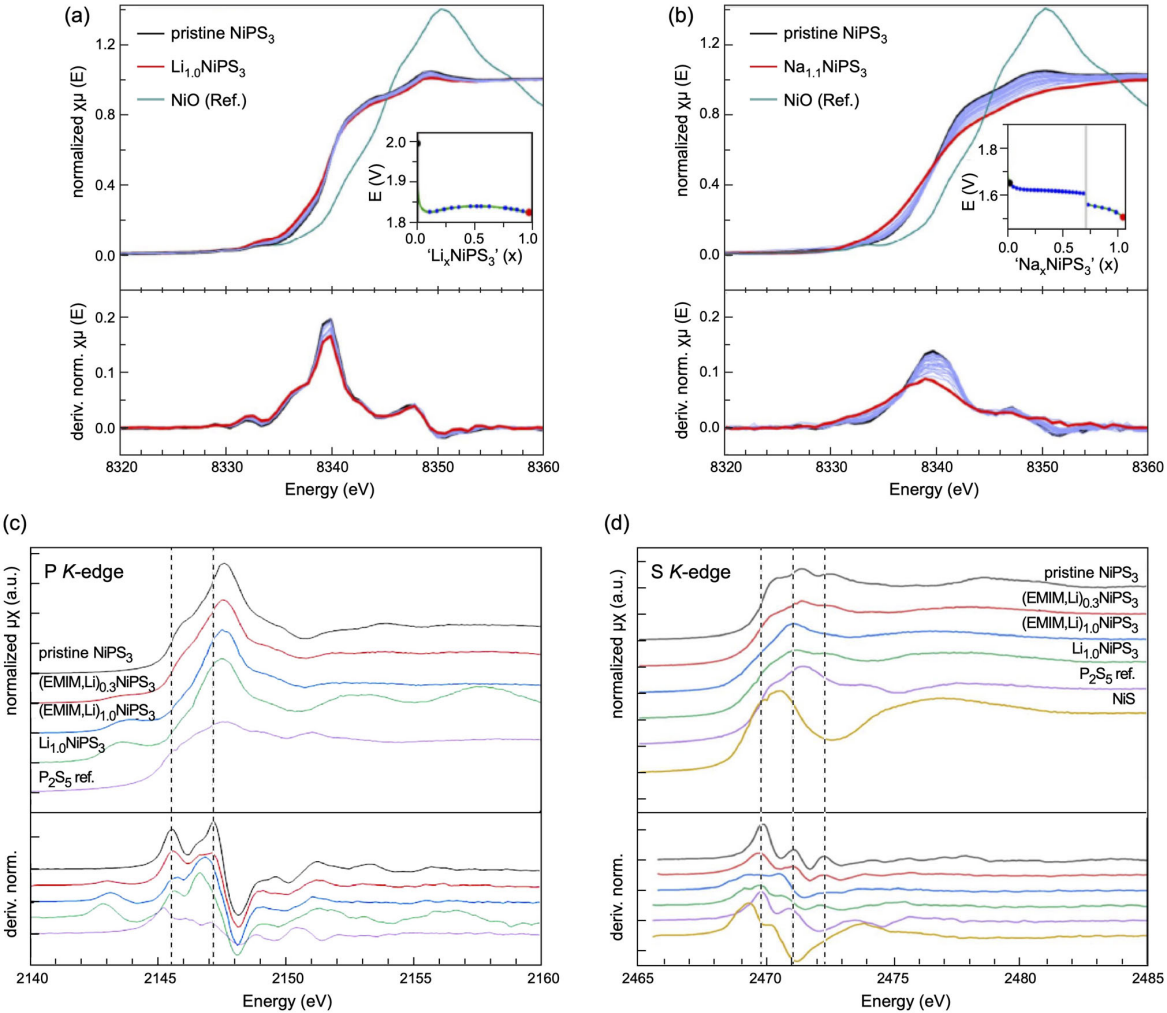
Operando XAS spectra of alkali ion intercalation into NiPS_3_: a) Ni *K*‐edge and dμ(E)/dE plots of Li_*x*_NiPS_3_ (0≤x≤1), b) Ni *K*‐edge and dμ(E)/dE plots of Na_*x*_NiPS_3_ (0≤x≤1.1). Insets show the corresponding potential profiles and the specific points of measurement. Ex situ XAS data: c) P *K*‐edge of pristine, EMIM and Li co‐intercalated NiPS_3_ and a P_2_S_5_ reference material including the first derivative of the normalized absorption coefficient, d) S *K*‐edge of pristine, EMIM and Li co‐intercalated NiPS_3_ and a P_2_S_5_ reference material including the first derivative of the normalized absorption coefficient. Reproduced with permission.^[^ ^106^ ^]^ Copyright 2024, The Royal Society of Chemistry.

Recent structural and spectroscopic investigations of Li and Na intercalation in NiPS_3_ have provided new insights into the intercalation mechanism and its electronic consequences^[^ ^106^ ^]^ (**Figure** **10**). These studies show that, during the initial stages of intercalation, up to ≈0.5 Li or Na ions per NiPS_3_ formula unit can be accommodated within the free octahedral sites located between the vdW‐bonded layers. Surprisingly, systematic in operando X‐ray absorption spectroscopy (XAS) measurements at the Ni *K*‐edge revealed minimal spectral changes over the range 0≤x≤1, indicating that Ni remains largely unaffected during this phase of Li insertion (see Figure 10a). A similar lack of significant spectral variation was also observed during Na intercalation (see Figure 10b). In contrast, clear modifications were detected at the P *K*‐ and S *K*‐edges, suggesting that the electron density is redistributed primarily on the (P_2_S_6_)^4−^ polyanion unit (see Figure 10c,d). From these observations, it was concluded that Li intercalation proceeds via an anionic redox mechanism in which Ni remains redox‐inactive. Conversely, Na intercalation induces a reduction of Ni, highlighting a cation‐specific intercalation behavior.^[^ ^106^ ^]^ Moreover, intercalation beyond 0.5 Li per NiPS_3_ formula unit leads to the formation of decomposition products, including Li_4_P_2_S_6_ as an intermediate and Li_2_S as the final product of lithiation.

In contrast to the electron transfer and host lattice reduction observed in NiPS_3_, the intercalation of Li, Na, and K into MnPS_3_ proceeds via ion transfer—specifically, a cation exchange mechanism between the guest ions and Mn^2+^ in the host lattice. This process results in the formation of Li(Na)(K)_2*x*_Mn_1–*x*_PS_3_ solid solutions.^[^ ^107^, ^108^, ^109^, ^110^ ^]^ The intercalation can be achieved through a simple soaking and stirring procedure, wherein MnPS_3_ is exposed to an aqueous LiCl, NaCl, or KCl solution. As found from the X‐ray diffraction, such intercalation of Li, Na, and K leads to the increase of the interlayer distance in Li(Na)(K)_2*x*_Mn_1–*x*_PS_3_ to $c^{\prime }=11.9$, 11.8, and 9.4 Å, respectively, compared to $c^{\prime }=6.5$ Å for the parent MnPS_3_ compound.^[^ ^108^, ^109^, ^110^ ^]^ XPS analysis of both pristine and intercalated compounds reveals no significant shifts in binding energy or changes in the spectral features—including the satellite structures—of the Mn core levels or the valence band. These observations strongly support a cation exchange intercalation mechanism rather than a redox process in MnPS_3_.

Recent advancements in the synthesis strategies of MPX_3_ materials have enabled the successful fabrication of the so‐called high‐entropy alloys (HEAs),^[^ ^111^, ^112^, ^113^, ^114^ ^]^ such as Co_0.6_(VMnNiZn)_0.4_PS_3_^[^ ^34^ ^]^ and FeMnNiVZnPS_3_^[^ ^33^ ^]^ (high‐resolution TEM images of these materials are shown in Figure 3). These materials are being actively explored for various energy‐related applications due to their unique structural and electrochemical properties. In the case of FeMnNiVZnPS_3_, the formation of aperiodic structures was observed throughout the crystals, resulting in a high density of strain soliton boundaries. These boundaries arise from the local stress imbalance caused by the presence of metal–sulfur (M–S) bonds of differing lengths and strengths. Theoretically predicted and experimentally confirmed through high‐resolution microscopy, these strain soliton boundaries serve as sites for localized electric field enhancement, which significantly promotes ion diffusion. Furthermore, the observed buckling of the vdW layers, in combination with these local electrostatic fields, was found to facilitate the diffusion of Na^+^ ions. The experimentally obtained Na^+^ diffusion coefficient for FeMnNiVZnPS_3_ is in the range of $10^{-9.7}-10^{-8.3}\;{\text{cm}}^{2}s^{-1}$, which is higher than the corresponding value of $10^{-10.6}-10^{-8.3}\;{\text{cm}}^{2}s^{-1}$ for FePS_3_.^[^ ^33^ ^]^ The HEA‐based electrodes fabricated from FeMnNiVZnPS_3_ also exhibit higher capacity of $733.2\;\;\text{mA}\;h\;g^{-1}$ compared to $647.9\;\;\text{mA}\;h\;g^{-1}$ for FePS_3_‐based electrodes. These experimental findings are supported by the respective theoretical modeling, which indicates a lower energy barrier (by ≈0.8 eV) for the Na^+^‐ions diffusion in FeMnNiVZnPS_3_ compared to FePS_3_. This insight is critical for the design and optimization of next‐generation battery materials based on high‐entropy vdW compounds.

Until recently, the magnetic properties of intercalation compounds based on MPX_3_ materials had not been extensively studied. Only with the emergence of modern theoretical insights into the magnetic exchange mechanisms in MPX_3_ lattices it has become possible to carry out more targeted investigations of these effects. In one of the earlier studies, the magnetic properties of MnPS_3_‐based intercalation compounds, with [M(salen)]^+^ complexes (M = Mn^3+^, Fe^3+^, Co^3+^; salen = *N*,*N*'‐ethylene‐bis(salicylideneimine)), were examined experimentally.^[^ ^115^ ^]^ The ion‐exchange mechanism characteristic for MnPX_3_ is also observed in this study, leading to creation of Mn vacancies in the formed intecalation compounds. It was found that the orientation of the intercalated molecules, as well as the resulting expansion of the interlayer vdW spacing, depended on the concentration of the guest species—likely affecting the doping level of individual MnPS_3_ layers. As a consequence of this dependence, intercalation with [Fe(salen)]^+^ and [Mn(salen)]^+^ led to the emergence of bulk spontaneous magnetization, with a transition from the paramagnetic to the ferromagnetic state observed at around 35 K. In contrast, intercalation with [Co(salen)]^+^ left the material in a paramagnetic state.

More intensive and systematic studies on the tuning of the magnetic properties of various MPX_3_ materials were motivated by recent theoretical works,^[^ ^20^, ^37^, ^82^, ^83^, ^84^, ^116^, ^117^, ^118^, ^119^ ^]^ which provided detailed analyses of their electronic and magnetic structures, along with potential modification strategies. Among the most natural and widely suggested approaches was the intercalation of Li into the MPX_3_ lattice, where Li acts as an electron donor, thereby tuning the magnetic exchange interactions between the originally antiferromagnetically coupled M^2+^ ions. This strategy was experimentally realized in ref. [120], where Li was electrochemically intercalated into bulk NiPS_3_, achieving a maximum composition of Li_*x*_NiPS_3_ with x=0.6 per formula unit. A corresponding expansion of the interlayer spacing was observed, confirming Li^+^ ion insertion into octahedral sites within the vdW gaps of NiPS_3_. As a result of the intercalation, the system exhibited an FM response at very low temperatures (≈2 K), while the original AFM ordering persisted at higher temperatures with a nearly unchanged Néel temperature of $T_{N}\approx 155$ K. This behavior was attributed to the zigzag AFM structure of the NiPS_3_ layers, consisting of two magnetic sublattices. Li intercalation introduces an imbalance in the spin‐up and spin‐down electron populations, rendering the magnetic moments of the two sublattices unequal and thus inducing weak ferromagnetism at low temperatures. This explanation is consistent with the small net magnetic moment of ≈$10^{-3}\;\mu B$ per Ni atom observed in the measurements.

**Figure 11:**
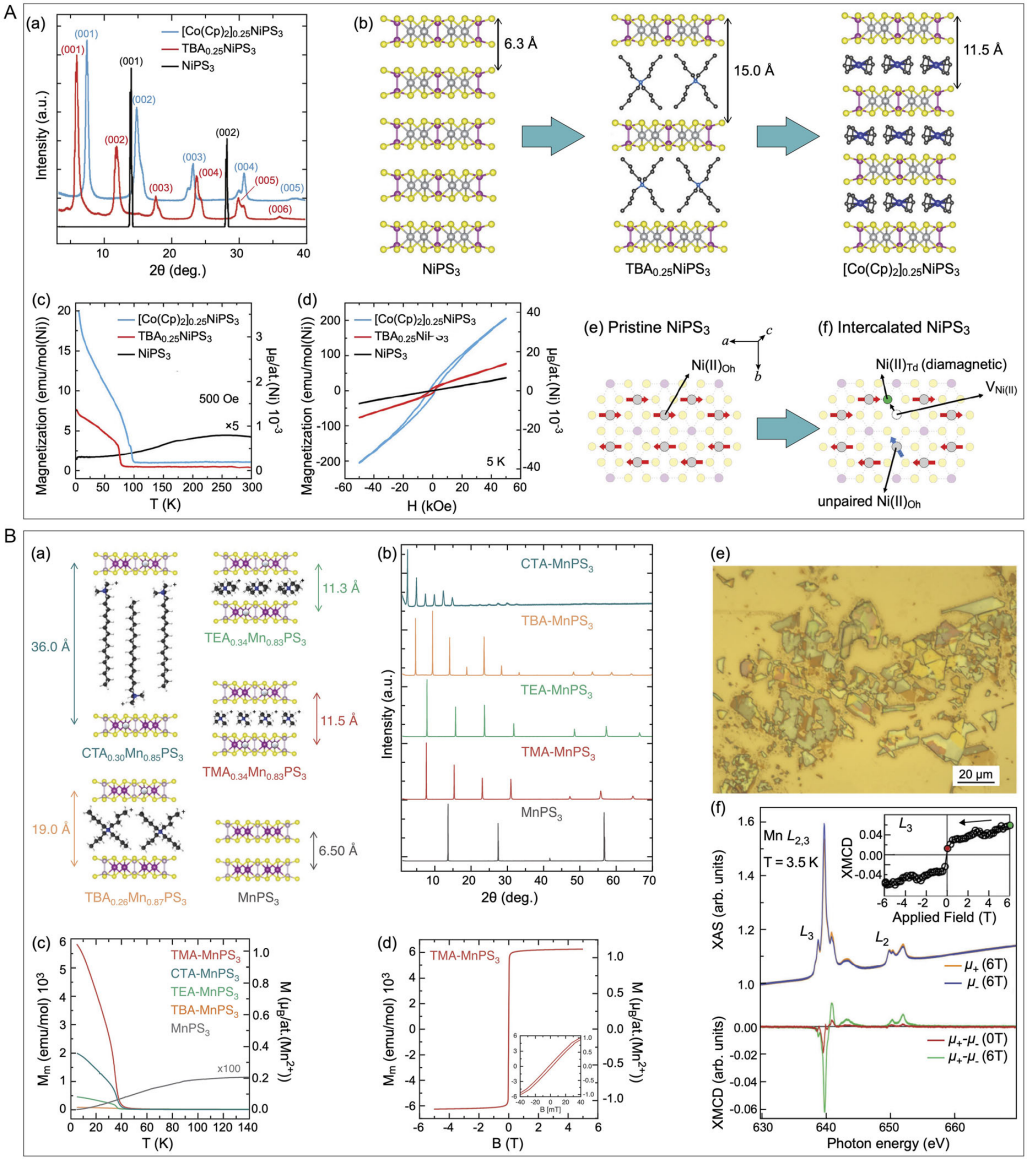
A) Experiments on intercalation of organic ions in bulk NiPS_3_: a) XRD patterns of bulk TBA_0.25_NiPS_3_ and [Co(Cp)_2_]_0.25_NiPS_3_ in comparison with those of bulk NiPS_3_; b) scheme of the intercalation and ion exchange when going from bulk NiPS_3_ through TBA_0.25_NiPS_3_ to [Co(Cp)_2_]_0.25_NiPS_3_. The respective interlayer distances are marked in the figure; c) field‐cooled molar magnetization versus temperature for bulk NiPS_3_, TBA_0.25_NiPS_3_, and [Co(Cp)_2_]_0.25_NiPS_3_ crystals. The applied field (500 Oe) is oriented parallel to the ab plane of the crystal; d) hysteresis loops at 5 K of bulk pristine NiPS_3_, TBA_0.25_NiPS_3_, and [Co(Cp)_2_]_0.25_NiPS_3_ crystals; e,f) Scheme of the structural change in the NiPS_3_ layer accompanying the reduction of the Ni atoms. Reproduced with permission.^[^ ^121^ ^]^ Copyright 2022, The Royal Society of Chemistry. B) Experiments on intercalation of organic ions in bulk MnPS_3_: a) schemes and b) respective XRD patterns of pristine bulk MnPS_3_ and four R_4_N^+^‐MnPS_3_ intercalates. The respective interlayer distances are marked in the figure; c) temperature dependence of the magnetization measured for a pristine MnPS_3_ crystal and for the four R_4_N^+^‐MnPS_3_ intercalates, under an out‐of‐plane magnetic field *B* = 0.1 T. The magnetization of the pristine MnPS_3_ crystal has been multiplied by a factor 100; d) hysteresis loop measured with the field applied out of plane for TMA‐MnPS_3_ intercalate. The inset displays the low field region, characterized by the opening of a magnetic hysteresis; e) optical image of the TMA‐MPS_3_‐intercalated flakes deposited on a gold substrate; f) top: Mn $L_{2,3}$ XAS for right (orange) and left (blue) circularly polarized light measured at 3.5 K and 6 T magnetic field (normal light incidence configuration). Inset: hysteresis loop of the XMCD intensities at the Mn $L_{3}$ edge. Bottom: XMCD signal ($\mu_{+}-\mu_{-}$) measured at 6 T applied field (green) and after field removal (red curve). Reproduced with permission.^[^ ^124^ ^]^ Copyright 2024, John Wiley and Sons.

Further studies on the intercalation of large molecules into MPX_3_ demonstrated the successful intercalation of tetrabutylammonium (TBA^+^) and tetraheptylammonium (THA^+^) cations into NiPS_3_ bulk^[^ ^121^, ^122^ ^]^ (**Figure** **11**A). After intercalating these species, transition temperatures between ferrimagnetic and AFM states were observed at 78 K for TBA^+^ and ≈100 K for THA^+^, with corresponding magnetic moments of $\approx 0.02-0.07\;\mu_{B}$ per unit cell. Further analysis revealed the reduction of Ni^2+^ ions to Ni^0^ upon intercalation, leading to a displacement of these atoms from the $O_{h}$ sites in the NiPS_3_ lattice to the $T_{d}$ sites. This displacement causes a significant change in the magnetic moment of Ni from the high‐spin state ($2.83\;\mu_{B}$) in $O_{h}$ to a low‐spin state (zero magnetic moment) in $T_{d}$. In the case of fractional intercalation, such as (TBA)_0.25_NiPS_3_, the AFM order of the parent NiPS_3_ lattice is not fully compensated, resulting in ferrimagnetic ordering in the intercalated compound. Theoretical analysis^[^ ^122^ ^]^ confirmed that at doping levels of 0.2−0.5 electrons per unit cell—achieved in the experiments—dopant electrons occupy only one Ni sublattice, leading to interchain spin splitting. This imbalance in the magnetic moments between the two Ni sublattices generates a net magnetic moment and ferrimagnetic ordering in the intercalated NiPS_3_. Interestingly, the initially intercalated TBA^+^ cations can be replaced by cobaltocene cations (Co(Cp)_2_^+^) through an ion‐exchange process, yielding a higher transition temperature of 98 K and a larger remanent magnetization^[^ ^121^ ^]^ (Figure 11A).

The intercalation of pyridine ions (PyH^+^) in FePS_3_ was achieved in ref. [123] using a one‐step ion‐exchange reaction, where the concentration of the intercalant was controlled by adjusting the temperature and intercalation duration. It was found that PyH^+^ ions can be arranged either parallel or perpendicular to the FePS_3_ layers, ultimately influencing the magnetic properties of the system. In both configurations, hard ferromagnetism was observed below a transition temperature of $T_{C}\approx 87$ K. However, the magnetic response was stronger when the PyH^+^ ions were aligned parallel to the FePS_3_ layers, suggesting a larger charge transfer between the guest and host materials in this phase. DFT calculations revealed that the intercalation of PyH^+^ significantly alters the $J_{n}$ coupling parameters, yielding values of $J_{1}=6.275$ meV, $J_{2}=-1.981$ meV, and $J_{3}=2.975$ meV. Along with a large magnetic moment for the Fe sites ($\approx 3.3-3.6\;\mu_{B}$) and FM ordering, these changes led to a predicted transition temperature of 35 K. Further experimental investigations ruled out the formation of Fe^2+^ defects (vacancies), indicating that the observed strong FM order in the PyH^+^‐intercalated FePS_3_ is due to pure electron doping.

While the previously discussed works demonstrate the appearance of (ferri)magnetic order in electron‐doped FePS_3_ and NiPS_3_ obtained through the intercalation of respective cations, a different mechanism for the emergence of magnetism in organic‐ion‐intercalated MnPS_3_ was recently discovered^[^ ^124^ ^]^ (Figure 11B). Various organic ions, including tetramethylammonium (TMA^+^), tetraethylammonium (TEA^+^), tetrabutylammonium (TBA^+^), and cetyltrimethylammonium (CTA^+^), were successfully intercalated into MnPS_3_ via the ion‐exchange mechanism, which was previously used for the intercalation of Li and K into MnPS_3_.^[^ ^107^, ^110^ ^]^ This process is nonredox and does not involve charge carrier doping in MnPS_3_. The final compositions determined experimentally were: TMA_0.34_Mn_0.83_PS_3_, TEA_0.34_Mn_0.83_PS_3_, TBA_0.26_Mn_0.87_PS_3_, and CTA_0.30_Mn_0.85_PS_3_. All of these intercalation systems clearly exhibit FM ordering at temperatures below $T_{C}\approx 40-60$ K, with the specific transition temperature varying depending on the intercalated species. The largest magnetic moment of $\approx 1.1\;\mu_{B}$ per Mn^2+^ was found for TMA_0.34_Mn_0.83_PS_3_, accompanied by a very narrow hysteresis loop. Theoretical modeling shows that Mn^2+^ vacancies are arranged within the AFM sublattice of MnPS_3_ with magnetic moments aligned in the same direction. This creates an imbalance between the two sublattices, leading to the observed magnetic state in the intercalated compound. Further investigation of the TMA‐MnPS_3_ intercalation compound using XMCD at very low temperatures revealed the presence of magnetic contrast in the remanent state, confirming the existence of an ordered FM state in the studied samples.

## 4 Conclusions and Outlooks

Layered vdW transition metal phosphorus trichalcogenides (MPX_3_) encompass a wide range of elements from the periodic table and can contain M^2+^ and M^3+^ magnetic ions with both filled and empty *d*‐shells. The metal ions are situated in octahedral coordination with chalcogen atoms, and the electronic and magnetic properties of these materials are strongly influenced by the specific M/X combination. In terms of electronic structure and properties, MPX_3_ materials exhibit a combination of three types of bonds—ionic, covalent, and vdW—making their property description a complex task, however, allowing for property tunability across a very wide range.

In this review, we provide an overview of the properties of the parent MPX_3_ materials and various approaches currently used to modify their electronic, optical, and magnetic characteristics. We also discuss the relationship between structural changes and electronic structure modifications. Among the methods highlighted for modifying the properties of vdW MPX_3_ materials are defect (vacancy) formation and its impact on material stability, adsorption of different species, and intercalation of various guest ions and molecules. As demonstrated, in many cases, the original properties—such as the wide‐bandgap and AFM ground state of the parent compounds—are significantly altered, resulting in new properties. These modifications open up opportunities to apply both the parent and modified MPX_3_ materials in areas such as catalysis, sensing, and spintronics. Notably, the most promising applications include using MPX_3_ in battery technology (for alkali‐metal ion storage) and in spintronics, as the AFM ground state can be converted into a FM state through the discussed modification approaches.

**Figure 12:**
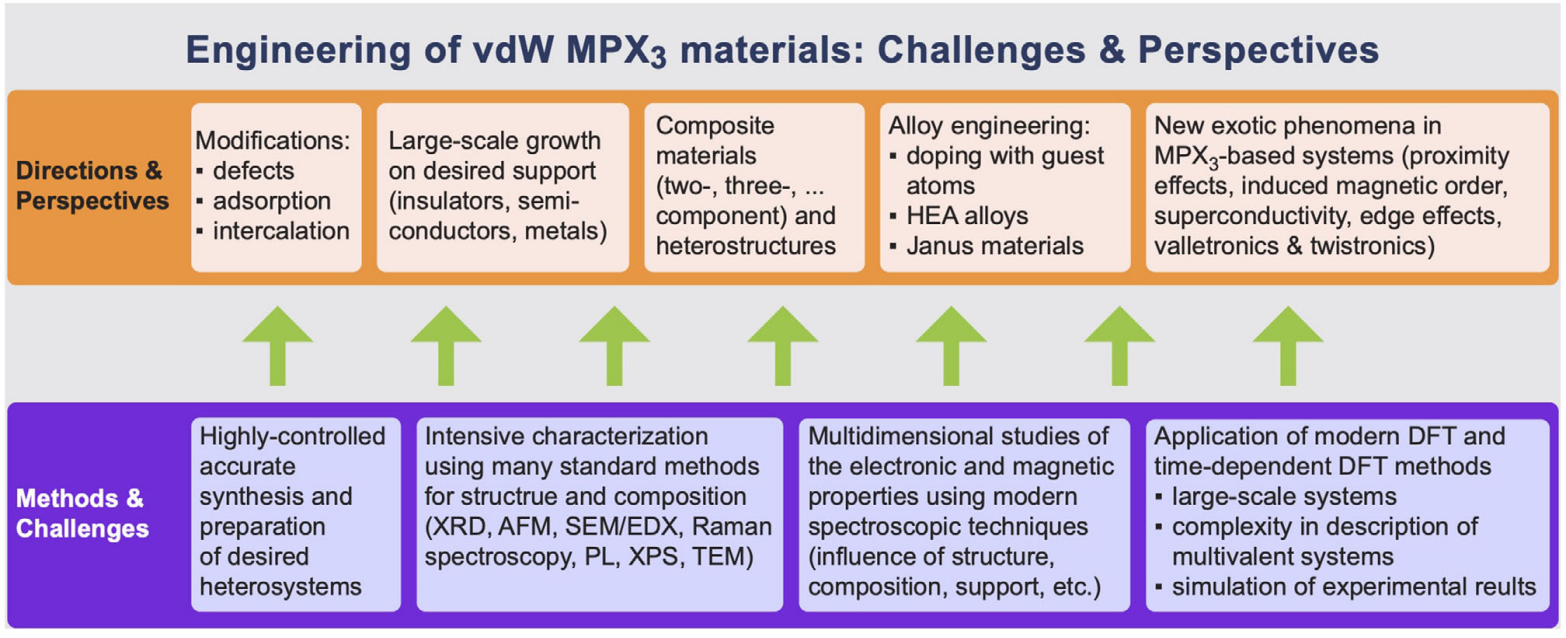
Challenges and perspectives in engineering of vdW MPX_3_ materials.

Despite significant advancements in the synthesis, modification, and proposed applications of various MPX_3_ materials and their derivatives, research on these materials—particularly regarding the fundamental approaches to studying their electronic and magnetic properties—remains in its early stages. Some challenges and potential short‐ to medium‐term prospects for MPX_3_ in various research and application areas can be outlined as follows (**Figure** **12**): 1) After the recent progress in the synthesis of high‐quality bulk MPX_3_ materials, it has become evident that their relatively large bandgaps and poor conductivity limit further studies and applications. One potential solution is the development of high‐quality, large‐scale ML or multilayer thin films of MPX_3_ on various substrates, including insulating, semiconducting, or metallic materials. As mentioned in Section 2, recent methods for synthesizing small ML‐thick MPX_3_ layers (with lateral sizes of ≈100  μm) on SiO_2_/Si substrates^[^ ^58^, ^59^ ^]^ show great potential, although scalability remains a significant challenge. Therefore, further improvements or new developments in this area are highly desirable. 2) The application of alloy engineering to modify and tune the electronic, optical, and magnetic properties of MPX_3_ materials is still limited and requires more systematic studies. Several directions can be considered: a) Doping of parent MPX_3_ compounds: Introducing small amounts of guest M^2+^ ions into the parent MPX_3_ compound could enhance properties for applications in catalysis or spintronics. Experimental and theoretical studies have shown that doping can significantly improve the catalytic properties of MPX_3_ for reactions like HER,^[^ ^86^ ^]^ and doping with elements having filled *d*‐shells can block the AFM interaction between neighboring M^2+^ ions, promoting a FM state.^[^ ^119^ ^]^ b) High‐entropy MPX_3_ alloys: A systematic and intensive theoretical analysis is needed to develop strategies for synthesizing high‐entropy MPX_3_ alloys with tailored properties.^[^ ^112^ ^]^ These alloys could have diverse applications, from catalysis and sensing to spintronics. c) Janus MPX_3_ materials: The hexagonal lattice of M^2+^ ions in MPX_3_ is sandwiched between oppositely oriented PX_3_ pyramids, leading to the concept of Janus MPX_3_ materials. These materials, where the top and bottom of the MPX_3_ layer are terminated with different chalcogens, have gained attention due to their unique properties, such as strong Rashba spin splitting, large piezoelectric effects, and enhanced catalytic performance. Although bulk MPX_3_ Janus compounds may not be feasible,^[^ ^125^, ^126^ ^]^ a promising strategy involves growing ML‐thick MPX_3_ on a desired support and replacing one layer of chalcogen atoms. This approach, successful in transition metal dichalcogenides, shows great potential for MPX_3_ Janus material synthesis.^[^ ^127^, ^128^ ^]^ 3) Detailed and reliable studies of the electronic structure of MPX_3_ and their derivatives remain scarce in the literature, often due to the challenges associated with the quality of the synthesized samples and potential charging effects caused by the wide bandgap. Time‐resolved spectroscopic studies are particularly valuable in this context, as they can provide new insights into the formation of metastable states in MPX_3_ materials. For example, such studies could help explore light‐induced FM‐to‐AFM transitions.^[^ ^129^, ^130^, ^131^ ^]^ Additionally, time‐resolved spectroscopic methods can be applied to investigate the mechanisms driving changes in the electronic, optical, and magnetic properties of MPX_3_ during modifications, as discussed in this review. This knowledge will be crucial for tailoring these materials to specific applications. 4) All of the aforementioned directions concerning the synthesis and experimental investigation of MPX_3_‐based systems must be accompanied by accurate and well‐founded theoretical studies. However, theoretical analysis of the structural, electronic and magnetic modifications in MPX_3_ through the discussed approaches remains a highly complex task.

First, any investigation of modified systems must be grounded in accurate modeling of the pristine compounds. This is particularly challenging in the case of MPX_3_, which can be considered a multivalent system featuring three types of bonding—covalent, ionic, and vdW.^[^ ^20^ ^]^ MPX_3_ materials also belong to the class of magnetic semiconductors, where accurate prediction of both the bandgap and their rich magnetic behavior can be particularly demanding. Consequently, a significant number of theoretical studies and database entries on MPX_3_ suffer from notable inaccuracies. These include incorrect predictions of the ground state (e.g., mistakenly identifying MPX_3_ as metallic or FM), incorrect crystallographic data leading to misidentified Brillouin zones, and inconsistencies between the presented DOS and band structures. Additionally, the use of nonphysical computational parameters—such as arbitrarily chosen Hubbard *U* values in DFT calculations—often leads to misleading results aimed at reproducing desired effects rather than reflecting physical reality.

Second, for all considered modifications—defects, adsorption, and intercalation—it is essential to construct appropriately sized supercells with realistic periodicities. This enables accurate modeling of experimental conditions, such as reasonable concentrations of defects, adsorbates, or intercalated species, and proper lattice matching in heterostructures. It is critical that: 1) all individual components of a heterostructure are correctly described independently,^[^ ^132^ ^]^ and 2) the chosen theoretical approaches consistently reproduce key experimental observables (e.g., lattice constants, bandgaps, magnetic moments) using a coherent set of computational parameters, including the exchange–correlation functional, *U* value, *k*‐point mesh, and dispersion correction scheme.

Finally, the modeling of structural and spectroscopic properties in MPX_3_‐based systems deserves special attention, particularly in the context of reproducibility. Structural characterization can benefit greatly from simulations of scanning probe microscopy data,^[^ ^133^ ^]^ which provide both atomic‐scale resolution and orbital‐specific insights. Given the strong correlation effects present in MPX_3_ compounds, the interpretation of spectroscopic data—such as XPS, NEXAFS, and time‐resolved photoemission^[^ ^53^ ^]^—offers valuable information on the electronic, optical, and magnetic properties of these materials and heterosystems. Particular focus should be placed on excitonic effects,^[^ ^134^, ^135^ ^]^ which are pronounced in MPX_3_ and represent a powerful tool for probing and understanding various phenomena in vdW heterostructures.

## Conflict of Interest

The authors declare no conflict of interest.

## Author Contributions

**Yuriy Dedkov**: conceptualization (equal); writing—original draft (equal); writing—review and editing (equal). **Elena Voloshina**: conceptualization (equal); writing—original draft (equal); writing—review and editing (equal).
